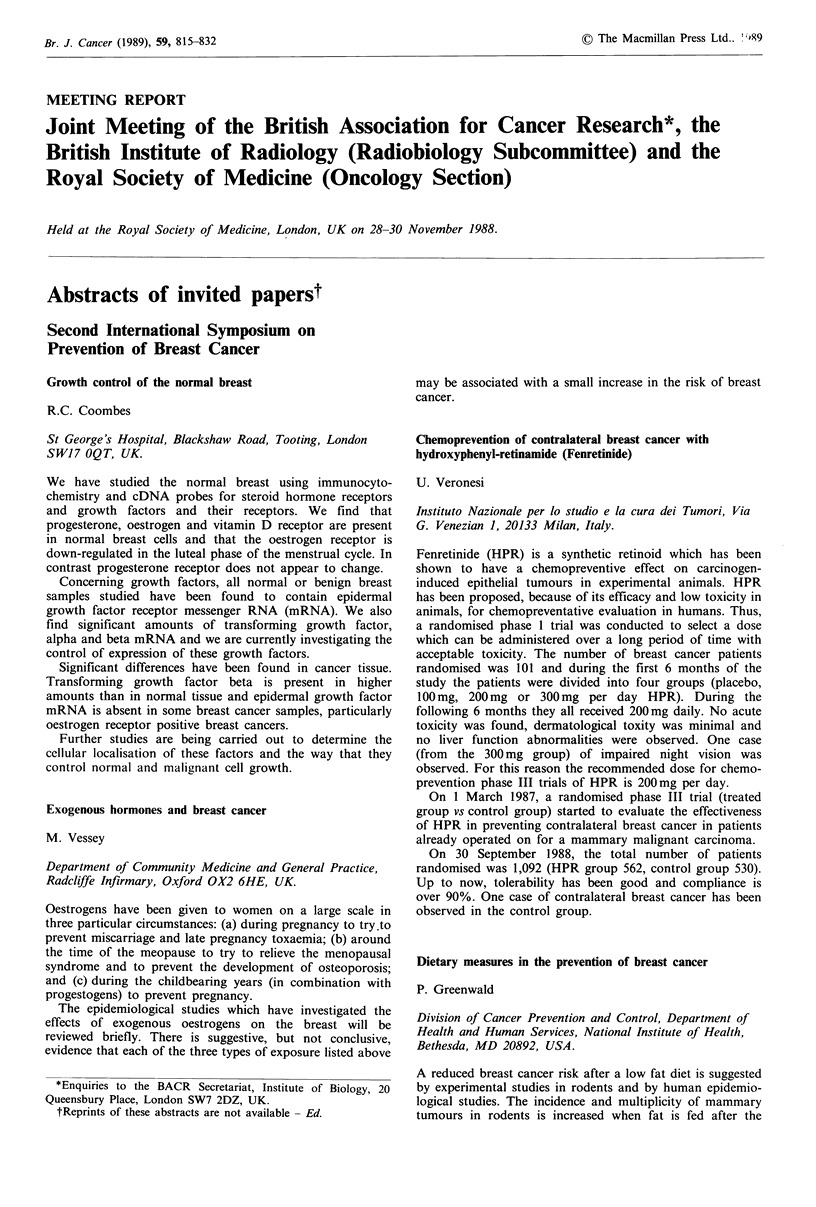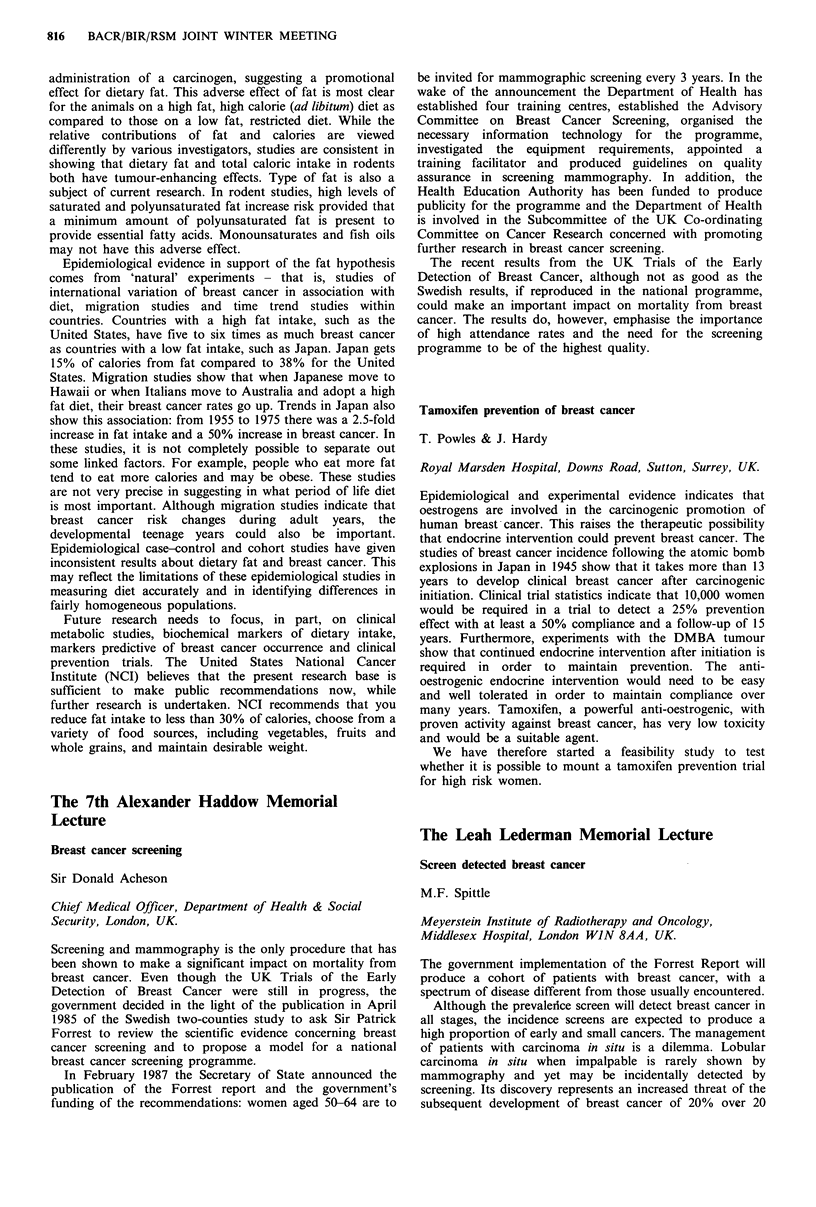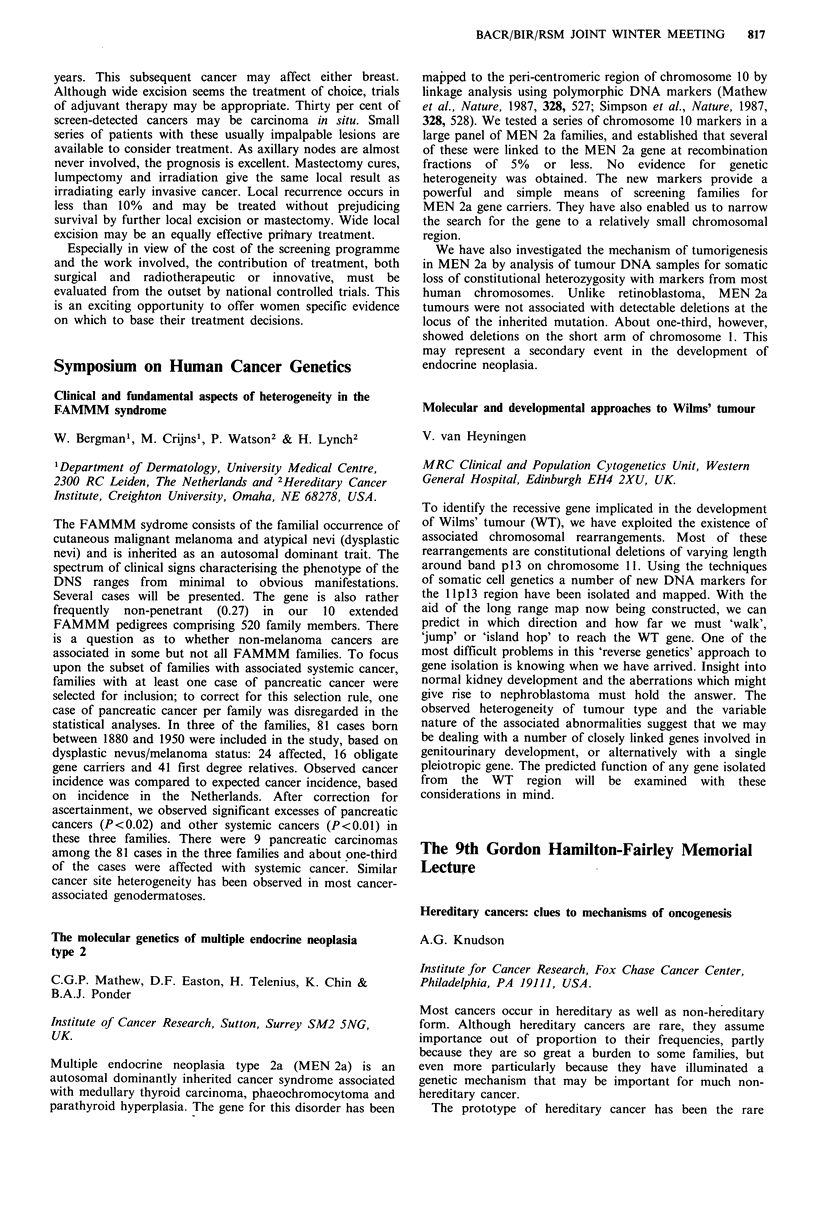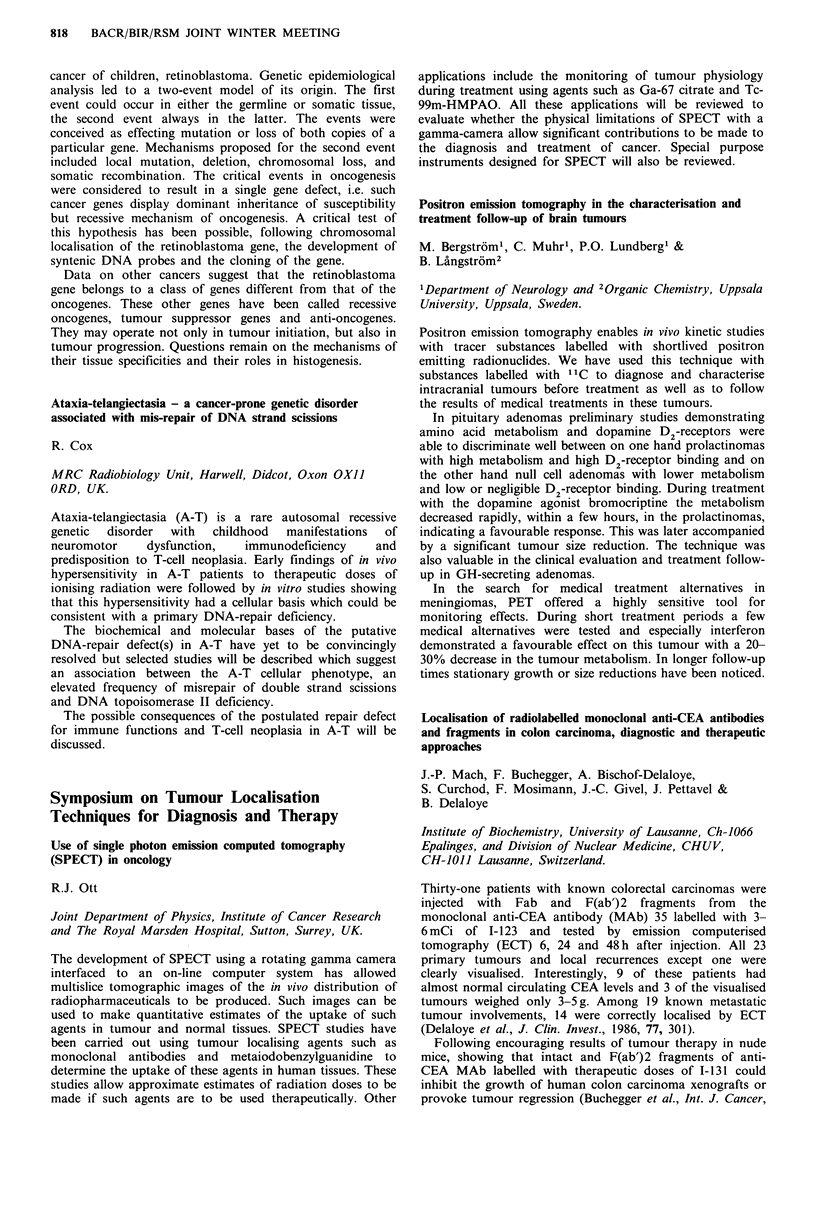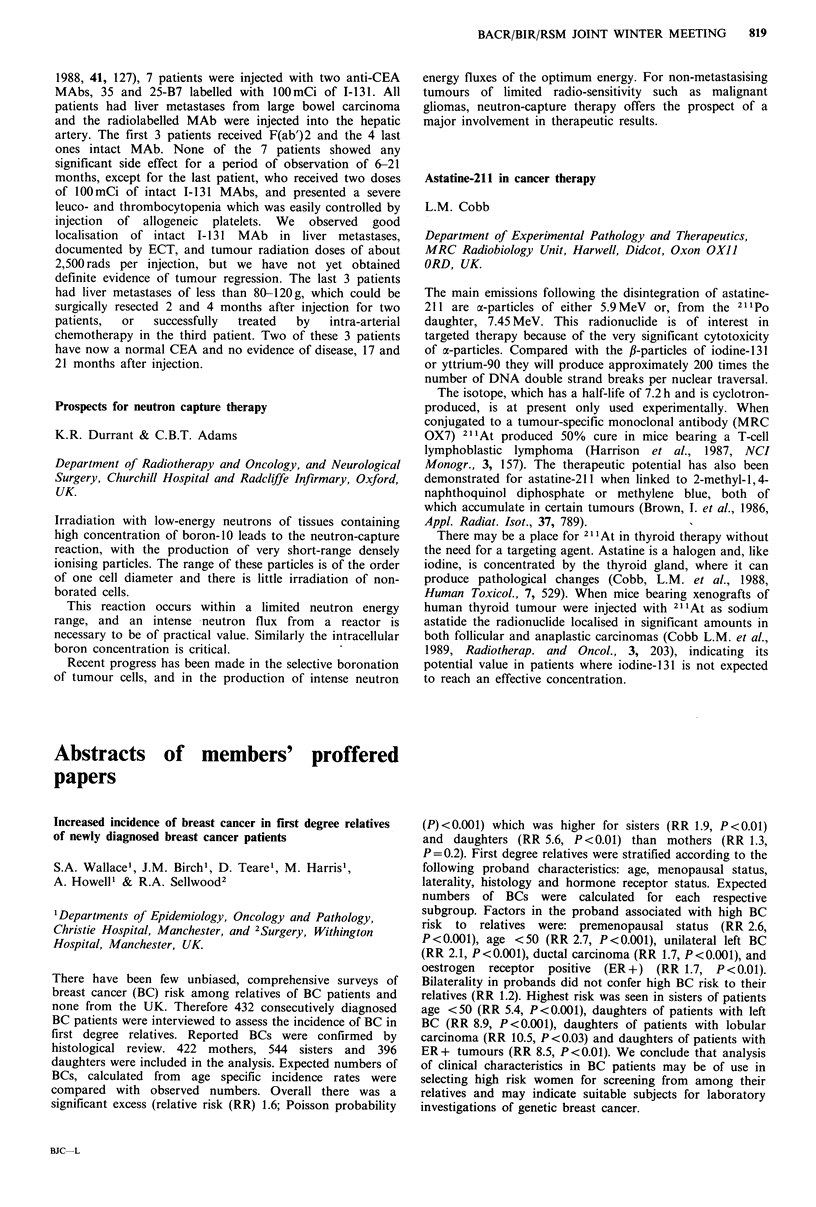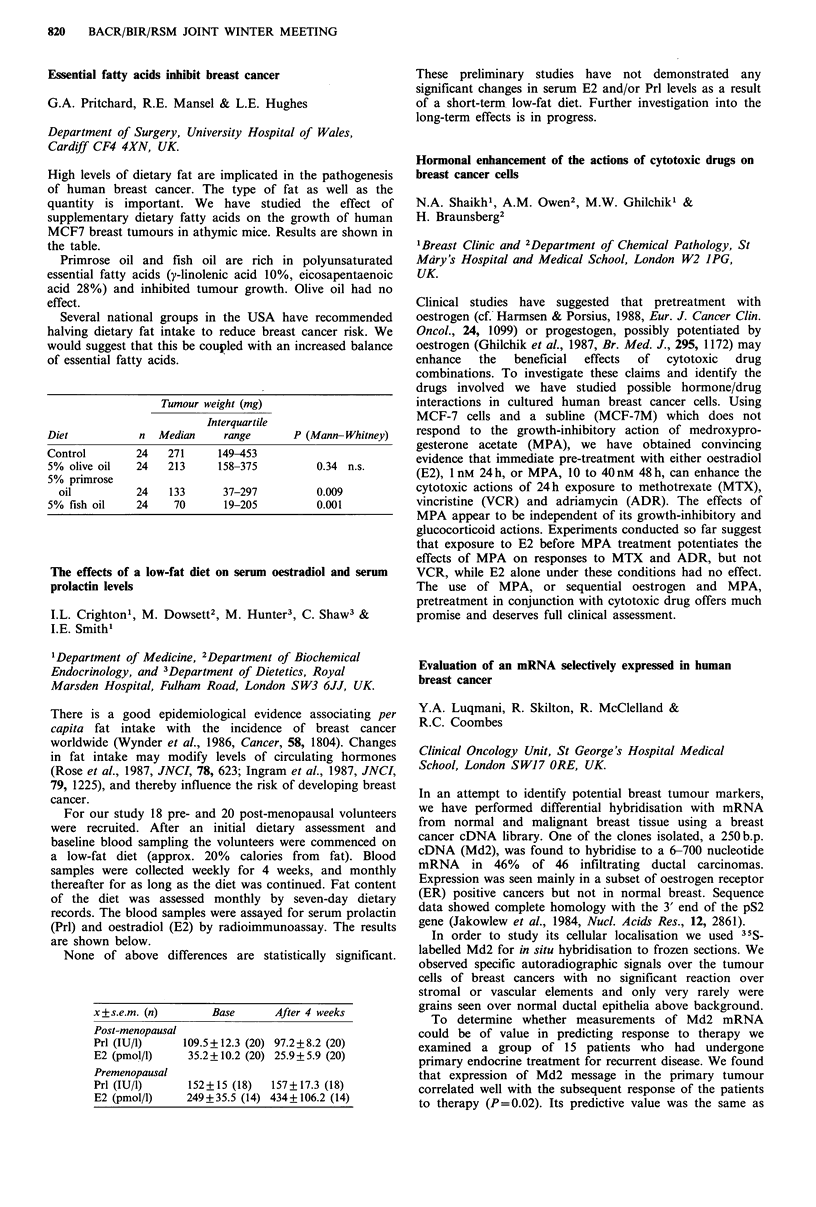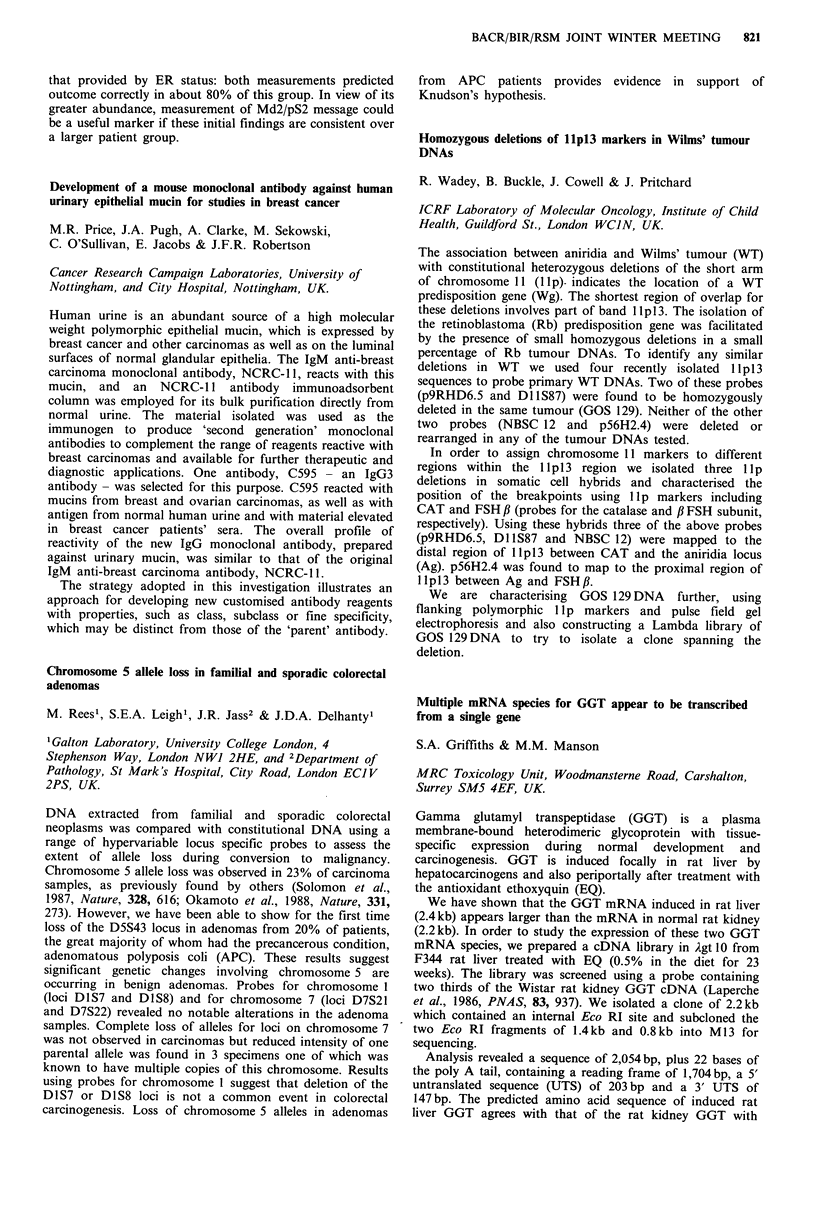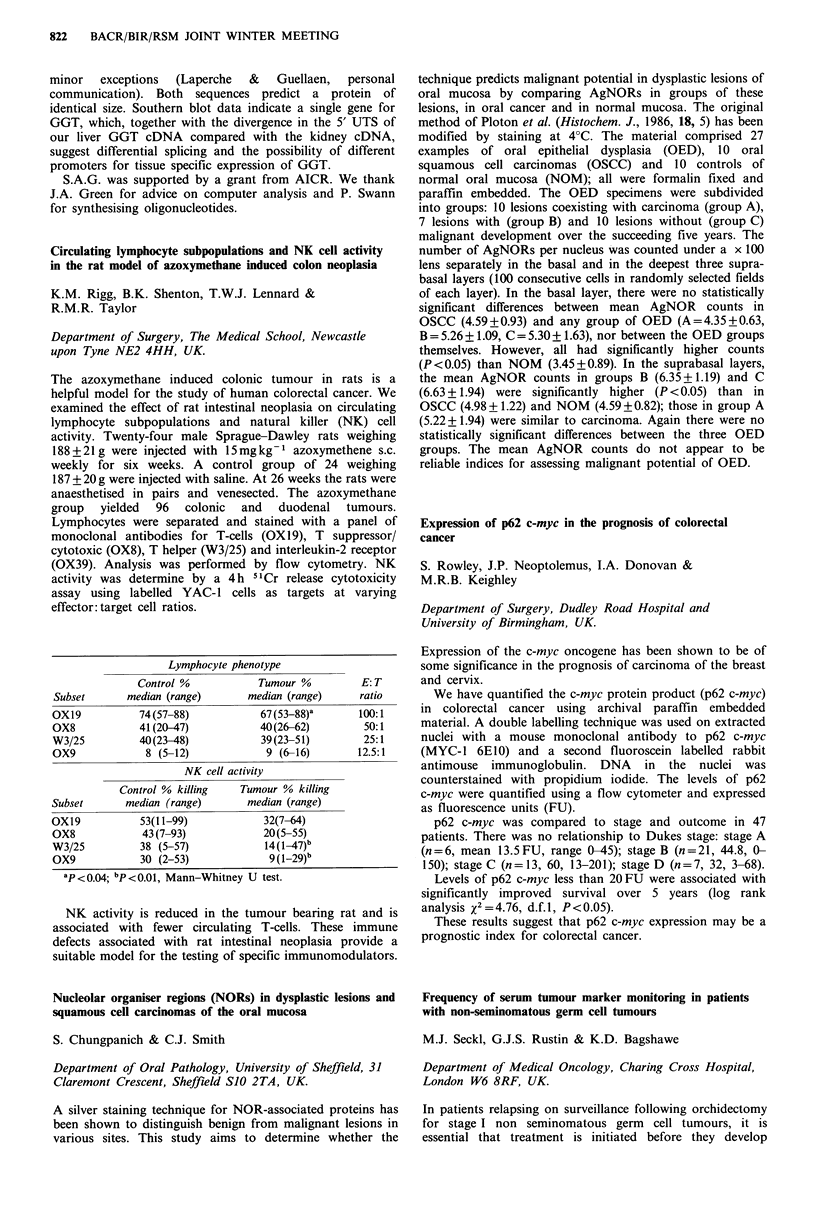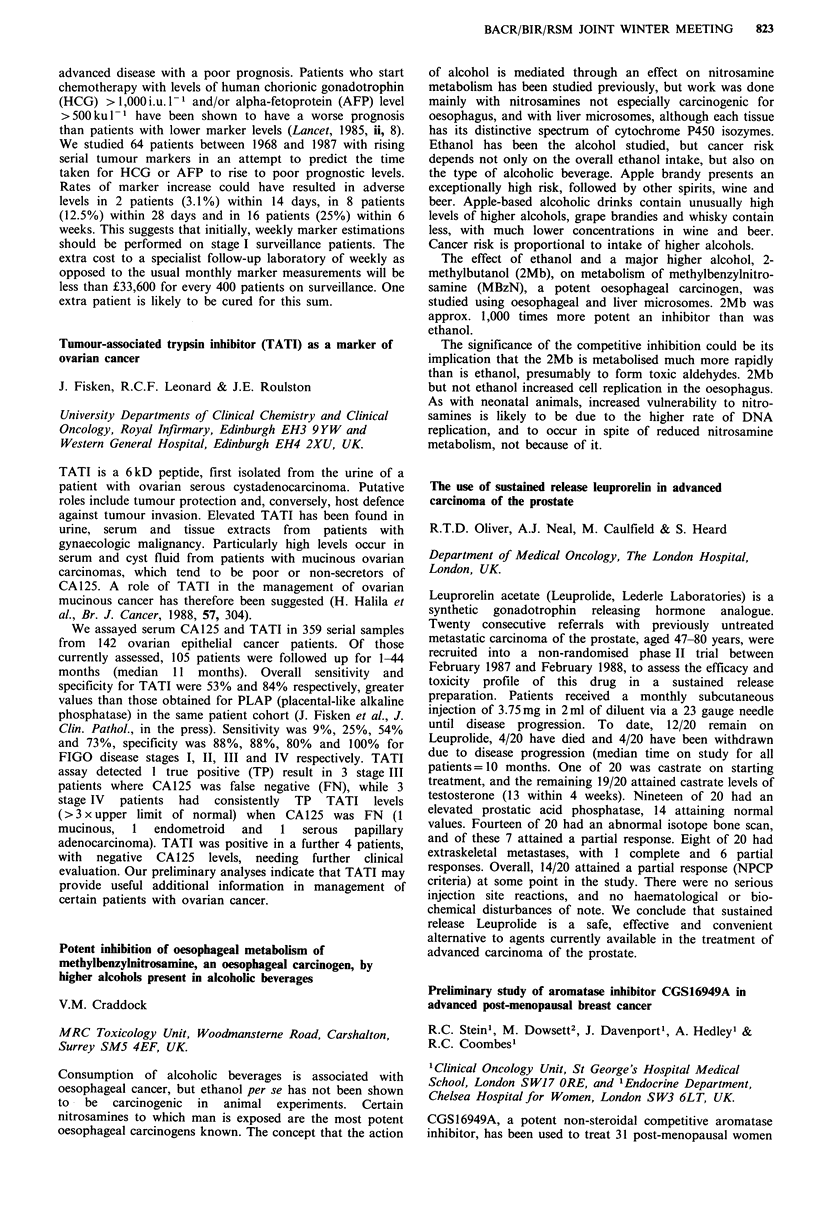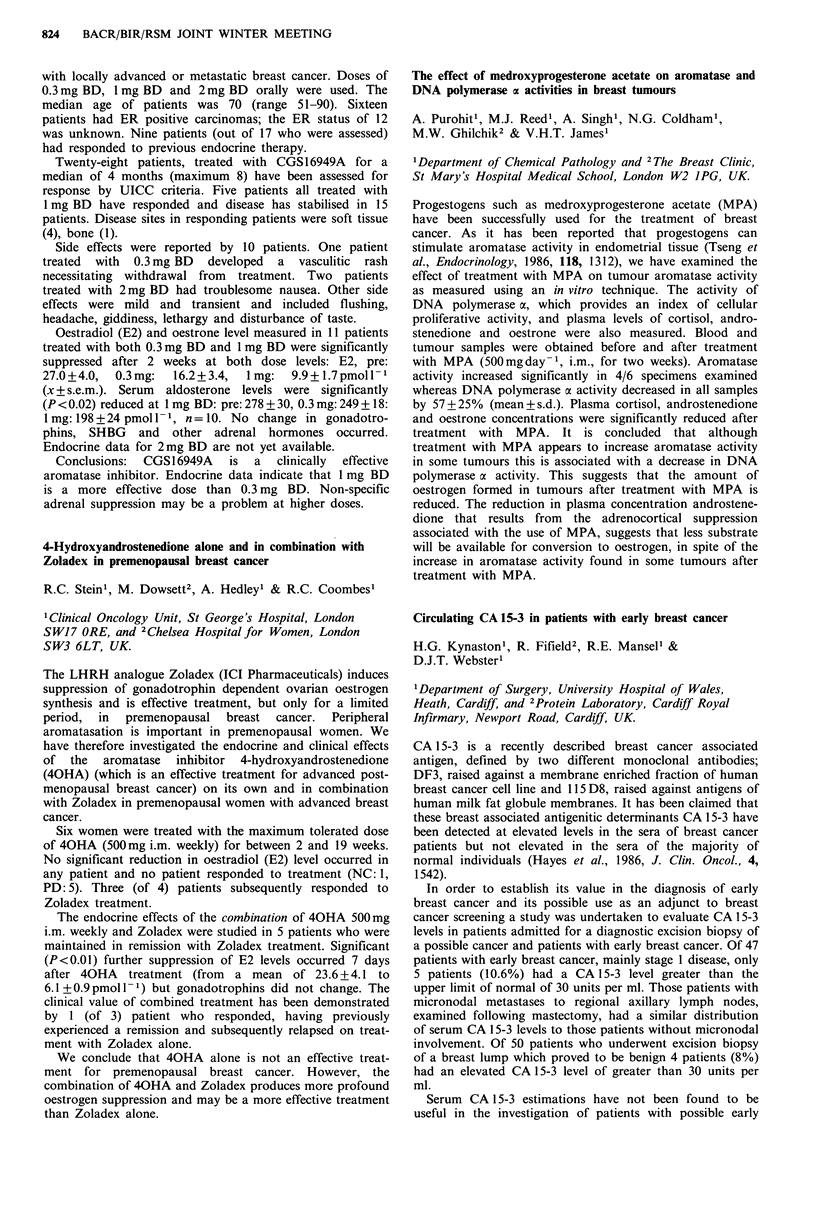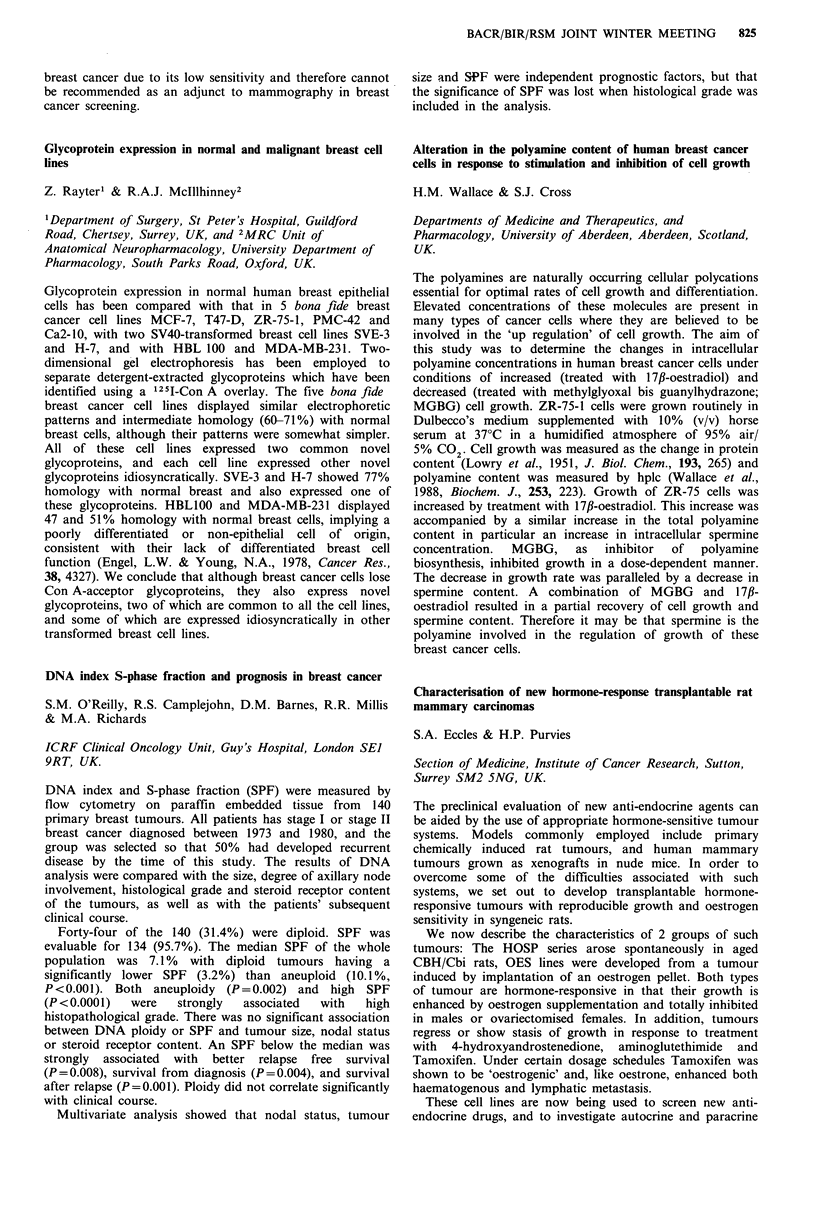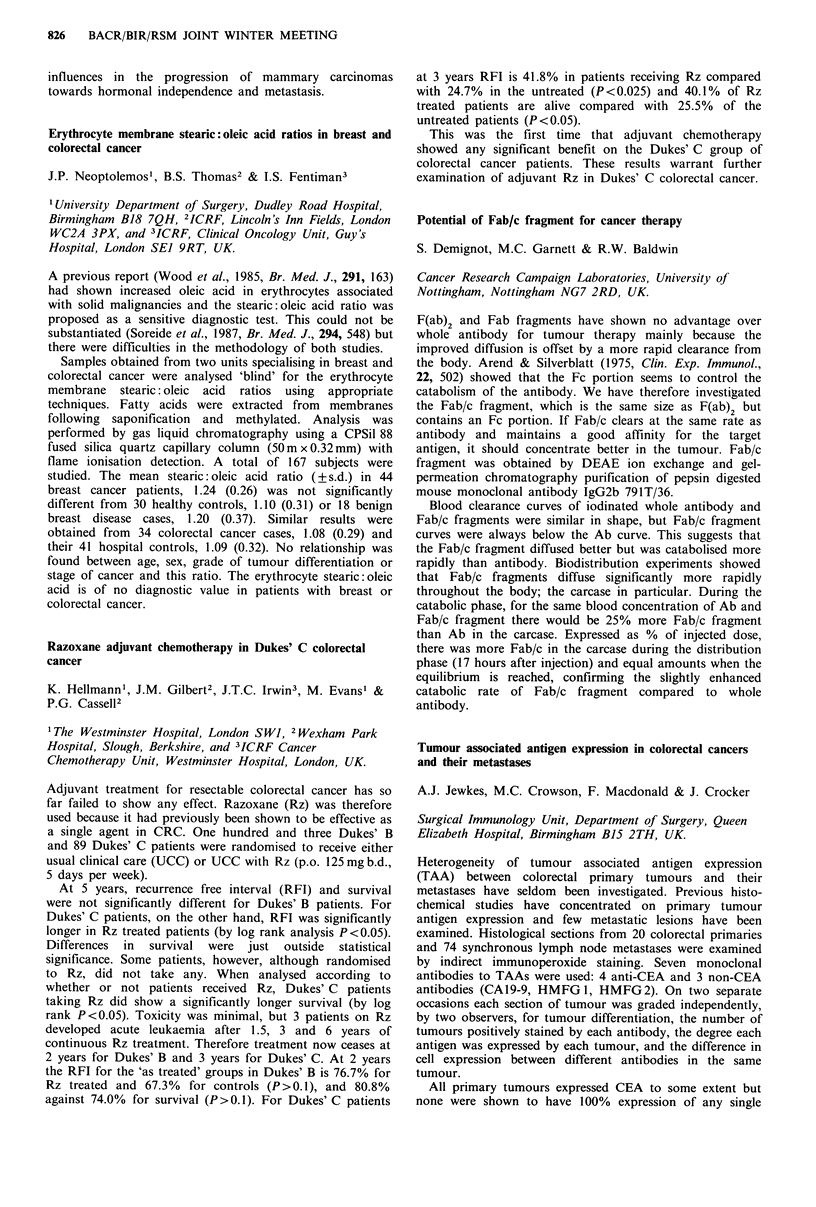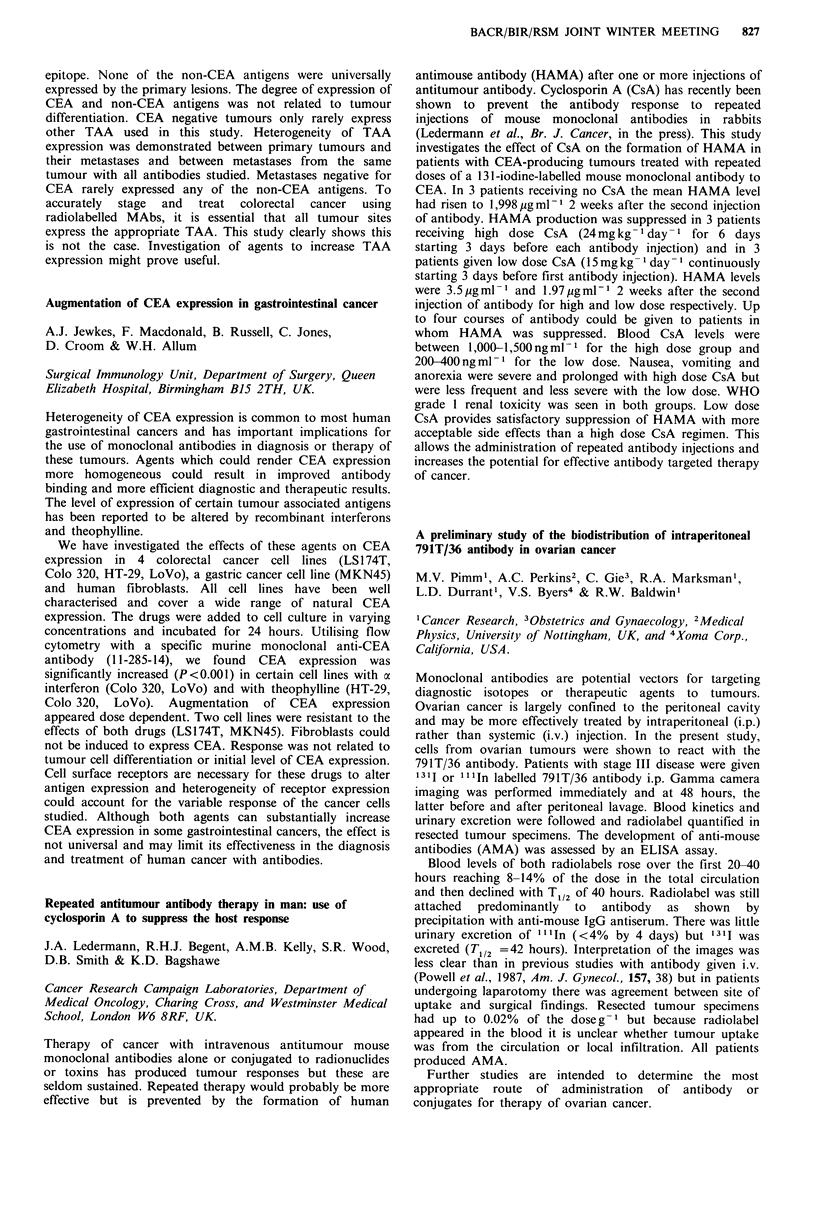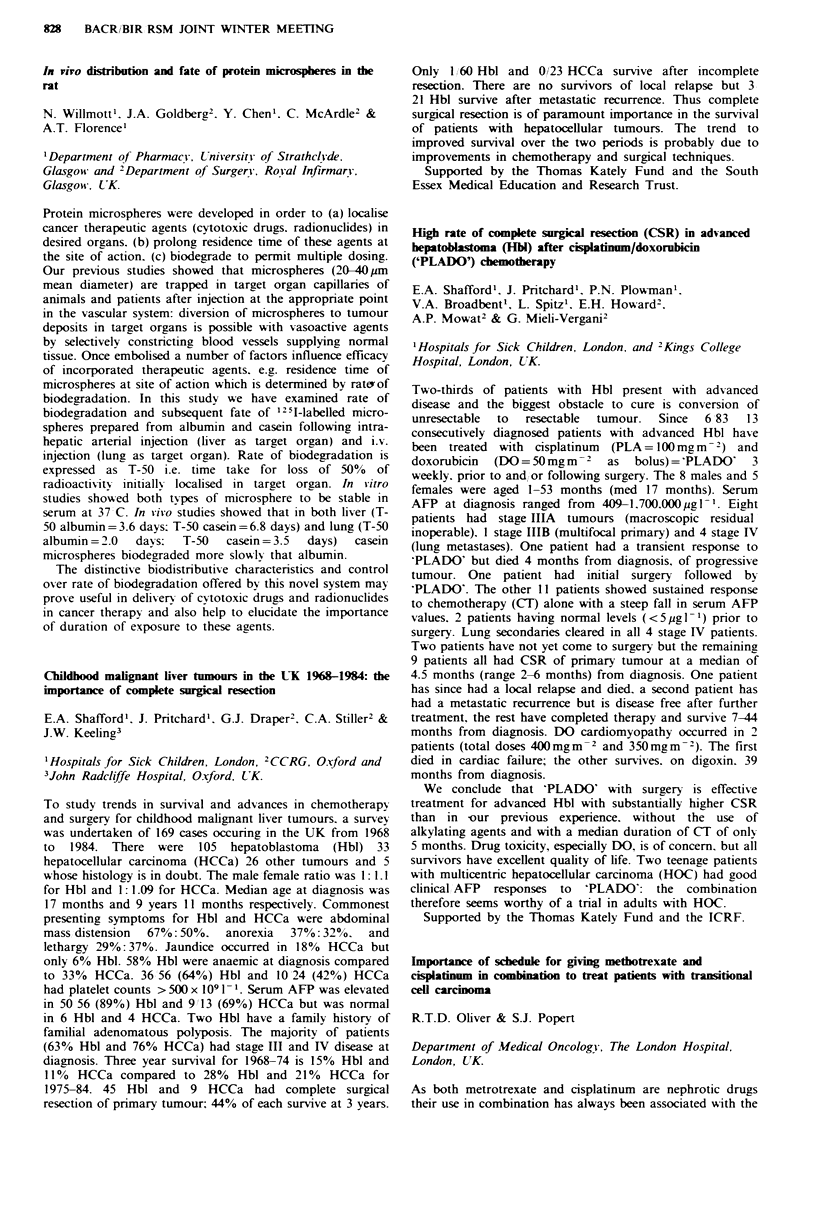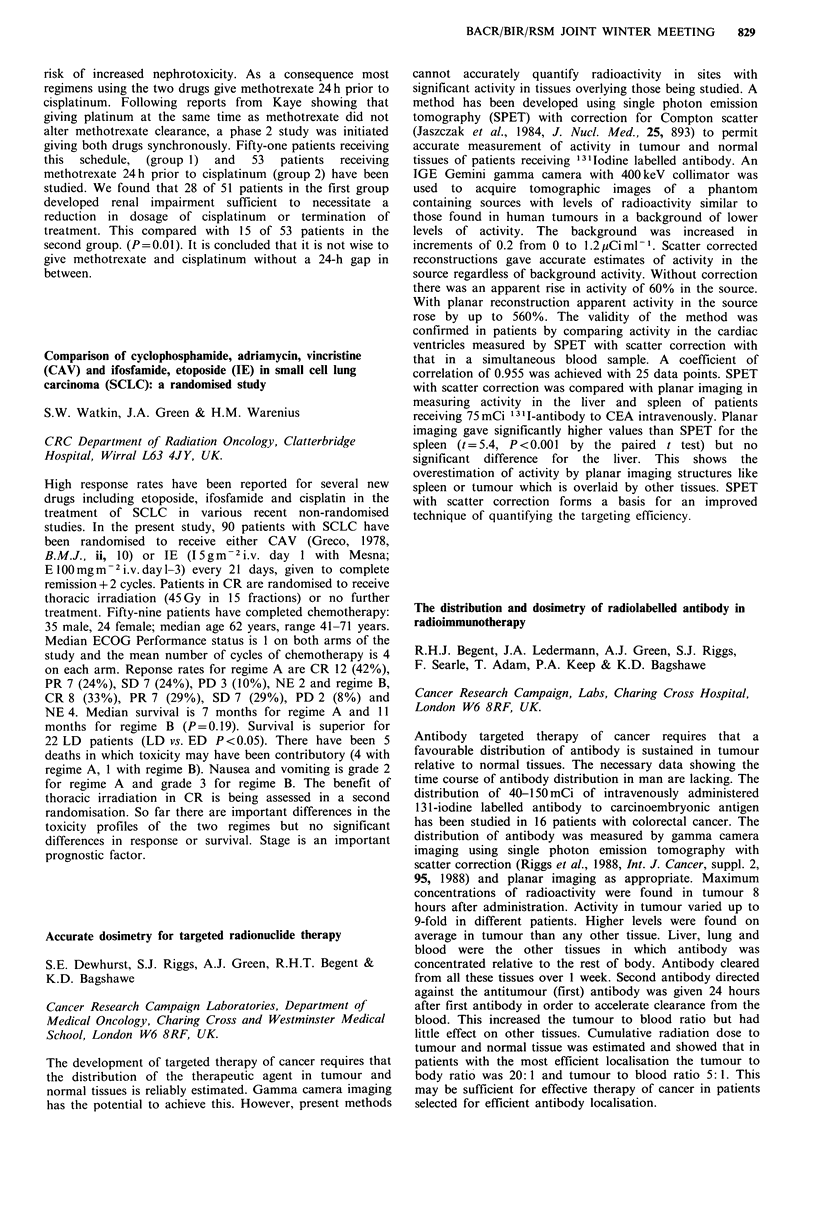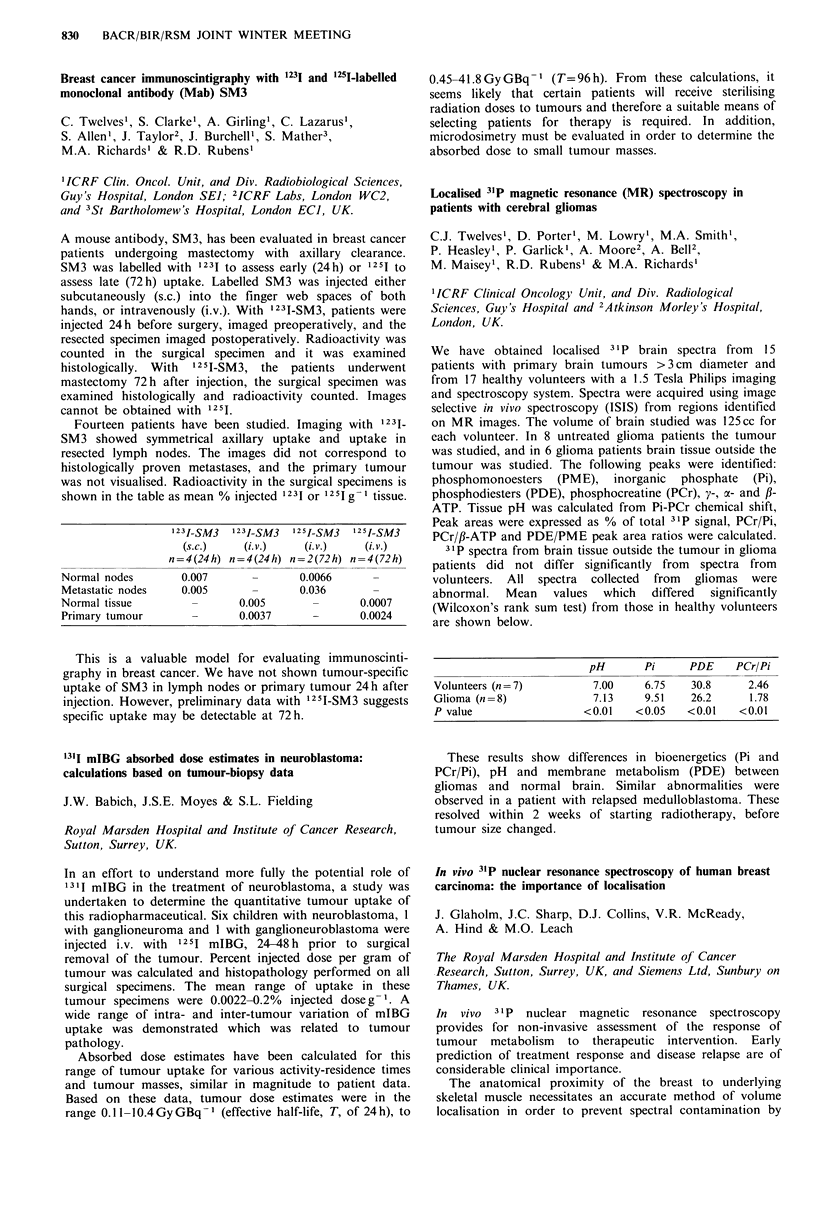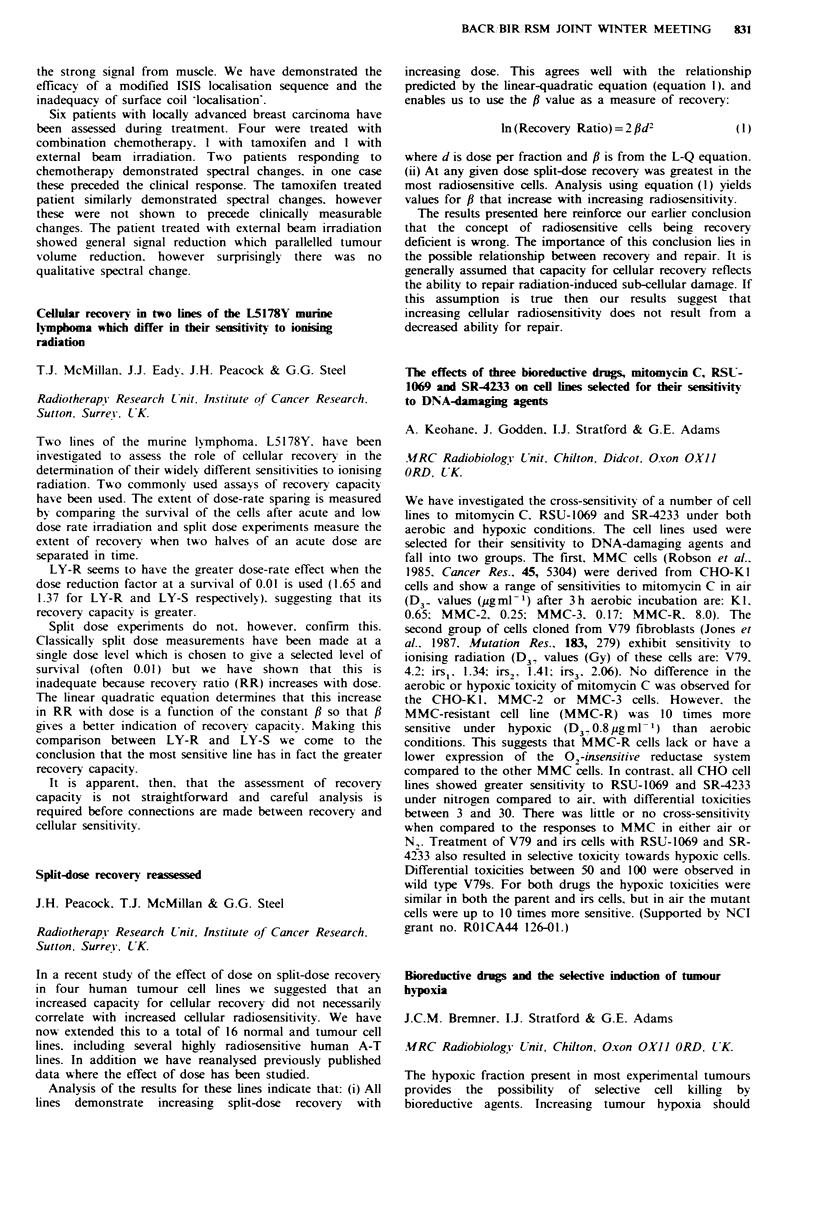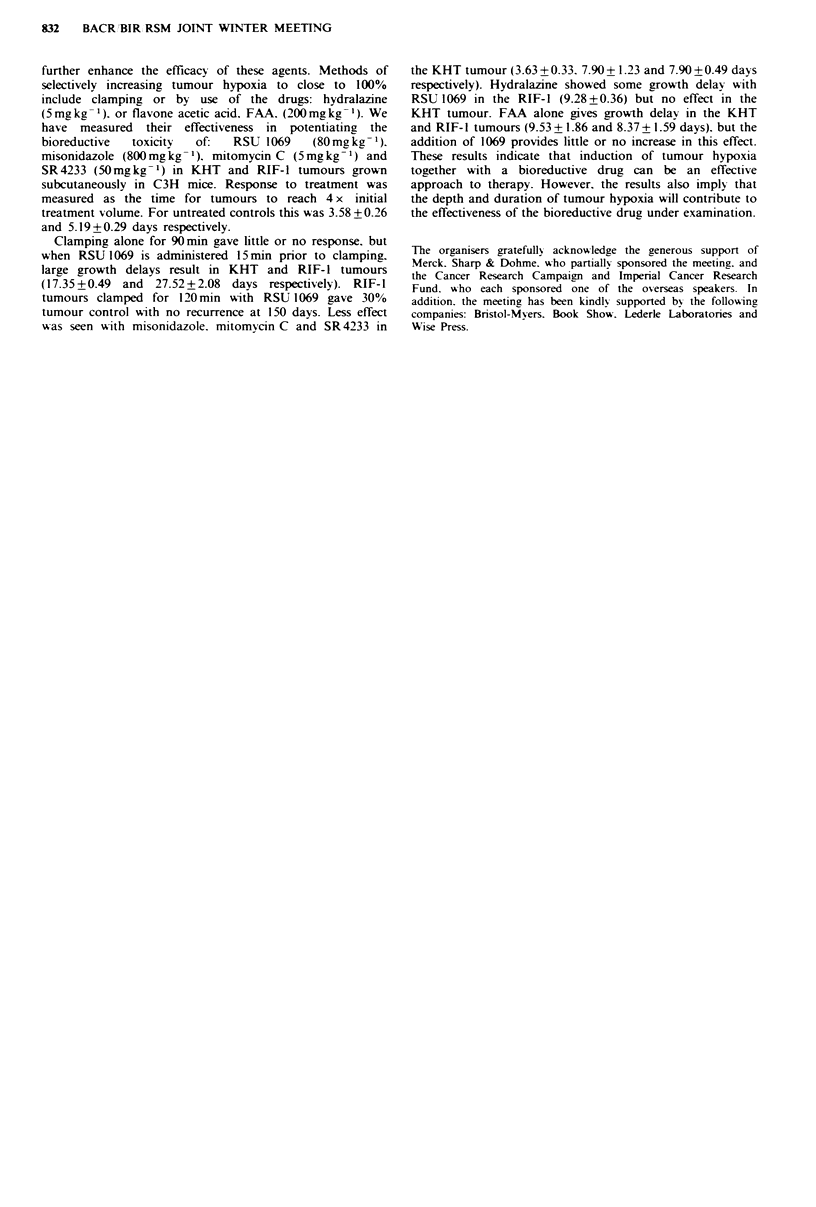# BACR/BIR/RSM joint winter meeting 28-30 November, 1988. Abstract of the proceedings

**Published:** 1989-05

**Authors:** 


Br. J. Cancer (1989), 59, 815–832 © The Macmillan Press Ltd., 1989

MEETING REPORT

### Joint Meeting of the British Association for Cancer Research*, the British Institute of Radiology (Radiobiology Subcommittee) and the Royal Society of Medicine (Oncology Section)

*Held at the Royal Society of Medicine, London, UK on 28–30 November 1988.*

### Abstracts of invited papers†

### Second International Symposium on Prevention of Breast Cancer

### Growth control of the normal breast

R.C. Coombes

*St George's Hospital, Blackshaw Road, Tooting, London SW17 0QT, UK.*

We have studied the normal breast using immunocytochemistry and cDNA probes for steroid hormone receptors and growth factors and their receptors. We find that progesterone, oestrogen and vitamin D receptor are present in normal breast cells and that the oestrogen receptor is down-regulated in the luteal phase of the menstrual cycle. In contrast progesterone receptor does not appear to change.

Concerning growth factors, all normal or benign breast samples studied have been found to contain epidermal growth factor receptor messenger RNA (mRNA). We also find significant amounts of transforming growth factor, alpha and beta mRNA and we are currently investigating the control of expression of these growth factors.

Significant differences have been found in cancer tissue. Transforming growth factor beta is present in higher amounts than in normal tissue and epidermal growth factor mRNA is absent in some breast cancer samples, particularly oestrogen receptor positive breast cancers.

Further studies are being carried out to determine the cellular localisation of these factors and the way that they control normal and malignant cell growth.

### Exogenous hormones and breast cancer

M. Vessey

*Department of Community Medicine and General Practice, Radcliffe Infirmary, Oxford OX2 6HE, UK.*

Oestrogens have been given to women on a large scale in three particular circumstances: (a) during pregnancy to try to prevent miscarriage and late pregnancy toxaemia; (b) around the time of the meopause to try to relieve the menopausal syndrome and to prevent the development of osteoporosis; and (c) during the childbearing years (in combination with progestogens) to prevent pregnancy.

The epidemiological studies which have investigated the effects of exogenous oestrogens on the breast will be reviewed briefly. There is suggestive, but not conclusive, evidence that each of the three types of exposure listed above may be associated with a small increase in the risk of breast cancer.

### Chemoprevention of contralateral breast cancer with hydroxyphenyl-retinamide (Fenretinide)

U. Veronesi

*Instituto Nazionale per lo studio e la cura dei Tumori, Via G. Venezian 1, 20133 Milan, Italy.*

Fenretinide (HPR) is a synthetic retinoid which has been shown to have a chemopreventive effect on carcinogen-induced epithelial tumours in experimental animals. HPR has been proposed, because of its efficacy and low toxicity in animals, for chemopreventative evaluation in humans. Thus, a randomised phase 1 trial was conducted to select a dose which can be administered over a long period of time with acceptable toxicity. The number of breast cancer patients randomised was 101 and during the first 6 months of the study the patients were divided into four groups (placebo, 100 mg, 200 mg or 300 mg per day HPR). During the following 6 months they all received 200 mg daily. No acute toxicity was found, dermatological toxity was minimal and no liver function abnormalities were observed. One case (from the 300 mg group) of impaired night vision was observed. For this reason the recommended dose for chemoprevention phase III trials of HPR is 200 mg per day.

On 1 March 1987, a randomised phase III trial (treated group *vs* control group) started to evaluate the effectiveness of HPR in preventing contralateral breast cancer in patients already operated on for a mammary malignant carcinoma.

On 30 September 1988, the total number of patients randomised was 1,092 (HPR group 562, control group 530). Up to now, tolerability has been good and compliance is over 90%. One case of contralateral breast cancer has been observed in the control group.

### Dietary measures in the prevention of breast cancer

P. Greenwald

*Division of Cancer Prevention and Control, Department of Health and Human Services, National Institute of Health, Bethesda, MD 20892, USA.*

A reduced breast cancer risk after a low fat diet is suggested by experimental studies in rodents and by human epidemiological studies. The incidence and multiplicity of mammary tumours in rodents is increased when fat is fed after the

*Enquiries to the BACR Secretariat, Institute of Biology, 20 Queensbury Place, London SW7 2DZ, UK.
†Reprints of these abstracts are not available – *Ed.*

administration of a carcinogen, suggesting a promotional effect for dietary fat. This adverse effect of fat is most clear for the animals on a high fat, high calorie (*ad libitum*) diet as compared to those on a low fat, restricted diet. While the relative contributions of fat and calories are viewed differently by various investigators, studies are consistent in showing that dietary fat and total caloric intake in rodents both have tumour-enhancing effects. Type of fat is also a subject of current research. In rodent studies, high levels of saturated and polyunsaturated fat increase risk provided that a minimum amount of polyunsaturated fat is present to provide essential fatty acids. Monounsaturates and fish oils may not have this adverse effect.

Epidemiological evidence in support of the fat hypothesis comes from 'natural' experiments – that is, studies of international variation of breast cancer in association with diet, migration studies and time trend studies within countries. Countries with a high fat intake, such as the United States, have five to six times as much breast cancer as countries with a low fat intake, such as Japan. Japan gets 15% of calories from fat compared to 38% for the United States. Migration studies show that when Japanese move to Hawaii or when Italians move to Australia and adopt a high fat diet, their breast cancer rates go up. Trends in Japan also show this association: from 1955 to 1975 there was a 2.5-fold increase in fat intake and a 50% increase in breast cancer. In these studies, it is not completely possible to separate out some linked factors. For example, people who eat more fat tend to eat more calories and may be obese. These studies are not very precise in suggesting in what period of life diet is most important. Although migration studies indicate that breast cancer risk changes during adult years, the developmental teenage years could also be important. Epidemiological case–control and cohort studies have given inconsistent results about dietary fat and breast cancer. This may reflect the limitations of these epidemiological studies in measuring diet accurately and in identifying differences in fairly homogeneous populations.

Future research needs to focus, in part, on clinical metabolic studies, biochemical markers of dietary intake, markers predictive of breast cancer occurrence and clinical prevention trials. The United States National Cancer Institute (NCI) believes that the present research base is sufficient to make public recommendations now, while further research is undertaken. NCI recommends that you reduce fat intake to less than 30% of calories, choose from a variety of food sources, including vegetables, fruits and whole grains, and maintain desirable weight.

### The 7th Alexander Haddow Memorial Lecture

### Breast cancer screening

Sir Donald Acheson

*Chief Medical Officer, Department of Health & Social Security, London, UK.*

Screening and mammography is the only procedure that has been shown to make a significant impact on mortality from breast cancer. Even though the UK Trials of the Early Detection of Breast Cancer were still in progress, the government decided in the light of the publication in April 1985 of the Swedish two-counties study to ask Sir Patrick Forrest to review the scientific evidence concerning breast cancer screening and to propose a model for a national breast cancer screening programme.

In February 1987 the Secretary of State announced the publication of the Forrest report and the government's funding of the recommendations: women aged 50–64 are to be invited for mammographic screening every 3 years. In the wake of the announcement the Department of Health has established four training centres, established the Advisory Committee on Breast Cancer Screening, organised the necessary information technology for the programme, investigated the equipment requirements, appointed a training facilitator and produced guidelines on quality assurance in screening mammography. In addition, the Health Education Authority has been funded to produce publicity for the programme and the Department of Health is involved in the Subcommittee of the UK Co-ordinating Committee on Cancer Research concerned with promoting further research in breast cancer screening.

The recent results from the UK Trials of the Early Detection of Breast Cancer, although not as good as the Swedish results, if reproduced in the national programme, could make an important impact on mortality from breast cancer. The results do, however, emphasise the importance of high attendance rates and the need for the screening programme to be of the highest quality.

### Tamoxifen prevention of breast cancer

T. Powles & J. Hardy

*Royal Marsden Hospital, Downs Road, Sutton, Surrey, UK.*

Epidemiological and experimental evidence indicates that oestrogens are involved in the carcinogenic promotion of human breast cancer. This raises the therapeutic possibility that endocrine intervention could prevent breast cancer. The studies of breast cancer incidence following the atomic bomb explosions in Japan in 1945 show that it takes more than 13 years to develop clinical breast cancer after carcinogenic initiation. Clinical trial statistics indicate that 10,000 women would be required in a trial to detect a 25% prevention effect with at least a 50% compliance and a follow-up of 15 years. Furthermore, experiments with the DMBA tumour show that continued endocrine intervention after initiation is required in order to maintain prevention. The anti-oestrogenic endocrine intervention would need to be easy and well tolerated in order to maintain compliance over many years. Tamoxifen, a powerful anti-oestrogenic, with proven activity against breast cancer, has very low toxicity and would be a suitable agent.

We have therefore started a feasibility study to test whether it is possible to mount a tamoxifen prevention trial for high risk women.

### The Leah Lederman Memorial Lecture

### Screen detected breast cancer

M.F. Spittle

*Meyerstein Institute of Radiotherapy and Oncology, Middlesex Hospital, London W1N 8AA, UK.*

The government implementation of the Forrest Report will produce a cohort of patients with breast cancer, with a spectrum of disease different from those usually encountered.

Although the prevalence screen will detect breast cancer in all stages, the incidence screens are expected to produce a high proportion of early and small cancers. The management of patients with carcinoma *in situ* is a dilemma. Lobular carcinoma *in situ* when impalpable is rarely shown by mammography and yet may be incidentally detected by screening. Its discovery represents an increased threat of the subsequent development of breast cancer of 20% over 20

years. This subsequent cancer may affect either breast. Although wide excision seems the treatment of choice, trials of adjuvant therapy may be appropriate. Thirty per cent of screen-detected cancers may be carcinoma *in situ*. Small series of patients with these usually impalpable lesions are available to consider treatment. As axillary nodes are almost never involved, the prognosis is excellent. Mastectomy cures, lumpectomy and irradiation give the same local result as irradiating early invasive cancer. Local recurrence occurs in less than 10% and may be treated without prejudicing survival by further local excision or mastectomy. Wide local excision may be an equally effective primary treatment.

Especially in view of the cost of the screening programme and the work involved, the contribution of treatment, both surgical and radiotherapeutic or innovative, must be evaluated from the outset by national controlled trials. This is an exciting opportunity to offer women specific evidence on which to base their treatment decisions.

### Symposium on Human Cancer Genetics

### Clinical and fundamental aspects of heterogeneity in the FAMMM syndrome

W. Bergman[1], M. Crijns[1], P. Watson[2] & H. Lynch[2]

[1]*Department of Dermatology, University Medical Centre, 2300 RC Leiden, The Netherlands and* [2]*Hereditary Cancer Institute, Creighton University, Omaha, NE 68278, USA.*

The FAMMM sydrome consists of the familial occurrence of cutaneous malignant melanoma and atypical nevi (dysplastic nevi) and is inherited as an autosomal dominant trait. The spectrum of clinical signs characterising the phenotype of the DNS ranges from minimal to obvious manifestations. Several cases will be presented. The gene is also rather frequently non-penetrant (0.27) in our 10 extended FAMMM pedigrees comprising 520 family members. There is a question as to whether non-melanoma cancers are associated in some but not all FAMMM families. To focus upon the subset of families with associated systemic cancer, families with at least one case of pancreatic cancer were selected for inclusion; to correct for this selection rule, one case of pancreatic cancer per family was disregarded in the statistical analyses. In three of the families, 81 cases born between 1880 and 1950 were included in the study, based on dysplastic nevus/melanoma status: 24 affected, 16 obligate gene carriers and 41 first degree relatives. Observed cancer incidence was compared to expected cancer incidence, based on incidence in the Netherlands. After correction for ascertainment, we observed significant excesses of pancreatic cancers ($P<0.02$) and other systemic cancers ($P<0.01$) in these three families. There were 9 pancreatic carcinomas among the 81 cases in the three families and about one-third of the cases were affected with systemic cancer. Similar cancer site heterogeneity has been observed in most cancer-associated genodermatoses.

### The molecular genetics of multiple endocrine neoplasia type 2

C.G.P. Mathew, D.F. Easton, H. Telenius, K. Chin & B.A.J. Ponder

*Institute of Cancer Research, Sutton, Surrey SM2 5NG, UK.*

Multiple endocrine neoplasia type 2a (MEN 2a) is an autosomal dominantly inherited cancer syndrome associated with medullary thyroid carcinoma, phaeochromocytoma and parathyroid hyperplasia. The gene for this disorder has been mapped to the peri-centromeric region of chromosome 10 by linkage analysis using polymorphic DNA markers (Mathew *et al., Nature,* 1987, **328,** 527; Simpson *et al., Nature,* 1987, **328,** 528). We tested a series of chromosome 10 markers in a large panel of MEN 2a families, and established that several of these were linked to the MEN 2a gene at recombination fractions of 5% or less. No evidence for genetic heterogeneity was obtained. The new markers provide a powerful and simple means of screening families for MEN 2a gene carriers. They have also enabled us to narrow the search for the gene to a relatively small chromosomal region.

We have also investigated the mechanism of tumorigenesis in MEN 2a by analysis of tumour DNA samples for somatic loss of constitutional heterozygosity with markers from most human chromosomes. Unlike retinoblastoma, MEN 2a tumours were not associated with detectable deletions at the locus of the inherited mutation. About one-third, however, showed deletions on the short arm of chromosome 1. This may represent a secondary event in the development of endocrine neoplasia.

### Molecular and developmental approaches to Wilms' tumour

V. van Heyningen

*MRC Clinical and Population Cytogenetics Unit, Western General Hospital, Edinburgh EH4 2XU, UK.*

To identify the recessive gene implicated in the development of Wilms' tumour (WT), we have exploited the existence of associated chromosomal rearrangements. Most of these rearrangements are constitutional deletions of varying length around band p13 on chromosome 11. Using the techniques of somatic cell genetics a number of new DNA markers for the 11p13 region have been isolated and mapped. With the aid of the long range map now being constructed, we can predict in which direction and how far we must 'walk', 'jump' or 'island hop' to reach the WT gene. One of the most difficult problems in this 'reverse genetics' approach to gene isolation is knowing when we have arrived. Insight into normal kidney development and the aberrations which might give rise to nephroblastoma must hold the answer. The observed heterogeneity of tumour type and the variable nature of the associated abnormalities suggest that we may be dealing with a number of closely linked genes involved in genitourinary development, or alternatively with a single pleiotropic gene. The predicted function of any gene isolated from the WT region will be examined with these considerations in mind.

### The 9th Gordon Hamilton-Fairley Memorial Lecture

### Hereditary cancers: clues to mechanisms of oncogenesis

A.G. Knudson

*Institute for Cancer Research, Fox Chase Cancer Center, Philadelphia, PA 19111, USA.*

Most cancers occur in hereditary as well as non-hereditary form. Although hereditary cancers are rare, they assume importance out of proportion to their frequencies, partly because they are so great a burden to some families, but even more particularly because they have illuminated a genetic mechanism that may be important for much non-hereditary cancer.

The prototype of hereditary cancer has been the rare cancer of children, retinoblastoma. Genetic epidemiological analysis led to a two-event model of its origin. The first event could occur in either the germline or somatic tissue, the second event always in the latter. The events were conceived as effecting mutation or loss of both copies of a particular gene. Mechanisms proposed for the second event included local mutation, deletion, chromosomal loss, and somatic recombination. The critical events in oncogenesis were considered to result in a single gene defect, i.e. such cancer genes display dominant inheritance of susceptibility but recessive mechanism of oncogenesis. A critical test of this hypothesis has been possible, following chromosomal localisation of the retinoblastoma gene, the development of syntenic DNA probes and the cloning of the gene.

Data on other cancers suggest that the retinoblastoma gene belongs to a class of genes different from that of the oncogenes. These other genes have been called recessive oncogenes, tumour suppressor genes and anti-oncogenes. They may operate not only in tumour initiation, but also in tumour progression. Questions remain on the mechanisms of their tissue specificities and their roles in histogenesis.

### Ataxia-telangiectasia – a cancer-prone genetic disorder associated with mis-repair of DNA strand scissions

R. Cox

*MRC Radiobiology Unit, Harwell, Didcot, Oxon OX11 0RD, UK.*

Ataxia-telangiectasia (A-T) is a rare autosomal recessive genetic disorder with childhood manifestations of neuromotor dysfunction, immunodeficiency and predisposition to T-cell neoplasia. Early findings of *in vivo* hypersensitivity in A-T patients to therapeutic doses of ionising radiation were followed by *in vitro* studies showing that this hypersensitivity had a cellular basis which could be consistent with a primary DNA-repair deficiency.

The biochemical and molecular bases of the putative DNA-repair defect(s) in A-T have yet to be convincingly resolved but selected studies will be described which suggest an association between the A-T cellular phenotype, an elevated frequency of misrepair of double strand scissions and DNA topoisomerase II deficiency.

The possible consequences of the postulated repair defect for immune functions and T-cell neoplasia in A-T will be discussed.

### Symposium on Tumour Localisation Techniques for Diagnosis and Therapy

### Use of single photon emission computed tomography (SPECT) in oncology

R.J. Ott

*Joint Department of Physics, Institute of Cancer Research and The Royal Marsden Hospital, Sutton, Surrey, UK.*

The development of SPECT using a rotating gamma camera interfaced to an on-line computer system has allowed multislice tomographic images of the *in vivo* distribution of radiopharmaceuticals to be produced. Such images can be used to make quantitative estimates of the uptake of such agents in tumour and normal tissues. SPECT studies have been carried out using tumour localising agents such as monoclonal antibodies and metaiodobenzylguanidine to determine the uptake of these agents in human tissues. These studies allow approximate estimates of radiation doses to be made if such agents are to be used therapeutically. Other applications include the monitoring of tumour physiology during treatment using agents such as Ga-67 citrate and Tc-99m-HMPAO. All these applications will be reviewed to evaluate whether the physical limitations of SPECT with a gamma-camera allow significant contributions to be made to the diagnosis and treatment of cancer. Special purpose instruments designed for SPECT will also be reviewed.

### Positron emission tomography in the characterisation and treatment follow-up of brain tumours

M. Bergström[1], C. Muhr[1], P.O. Lundberg[1] & B. Långström[2]

[1]*Department of Neurology and* [2]*Organic Chemistry, Uppsala University, Uppsala, Sweden.*

Positron emission tomography enables *in vivo* kinetic studies with tracer substances labelled with shortlived positron emitting radionuclides. We have used this technique with substances labelled with $^{11}C$ to diagnose and characterise intracranial tumours before treatment as well as to follow the results of medical treatments in these tumours.

In pituitary adenomas preliminary studies demonstrating amino acid metabolism and dopamine $D_2$-receptors were able to discriminate well between on one hand prolactinomas with high metabolism and high $D_2$-receptor binding and on the other hand null cell adenomas with lower metabolism and low or negligible $D_2$-receptor binding. During treatment with the dopamine agonist bromocriptine the metabolism decreased rapidly, within a few hours, in the prolactinomas, indicating a favourable response. This was later accompanied by a significant tumour size reduction. The technique was also valuable in the clinical evaluation and treatment follow-up in GH-secreting adenomas.

In the search for medical treatment alternatives in meningiomas, PET offered a highly sensitive tool for monitoring effects. During short treatment periods a few medical alternatives were tested and especially interferon demonstrated a favourable effect on this tumour with a 20–30% decrease in the tumour metabolism. In longer follow-up times stationary growth or size reductions have been noticed.

### Localisation of radiolabelled monoclonal anti-CEA antibodies and fragments in colon carcinoma, diagnostic and therapeutic approaches

J.-P. Mach, F. Buchegger, A. Bischof-Delaloye, S. Curchod, F. Mosimann, J.-C. Givel, J. Pettavel & B. Delaloye

*Institute of Biochemistry, University of Lausanne, Ch-1066 Epalinges, and Division of Nuclear Medicine, CHUV, CH-1011 Lausanne, Switzerland.*

Thirty-one patients with known colorectal carcinomas were injected with Fab and F(ab′)2 fragments from the monoclonal anti-CEA antibody (MAb) 35 labelled with 3–6 mCi of I-123 and tested by emission computerised tomography (ECT) 6, 24 and 48 h after injection. All 23 primary tumours and local recurrences except one were clearly visualised. Interestingly, 9 of these patients had almost normal circulating CEA levels and 3 of the visualised tumours weighed only 3–5 g. Among 19 known metastatic tumour involvements, 14 were correctly localised by ECT (Delaloye *et al.*, *J. Clin. Invest.*, 1986, **77**, 301).

Following encouraging results of tumour therapy in nude mice, showing that intact and F(ab′)2 fragments of anti-CEA MAb labelled with therapeutic doses of I-131 could inhibit the growth of human colon carcinoma xenografts or provoke tumour regression (Buchegger *et al.*, *Int. J. Cancer*,

1988, **41**, 127), 7 patients were injected with two anti-CEA MAbs, 35 and 25-B7 labelled with 100 mCi of I-131. All patients had liver metastases from large bowel carcinoma and the radiolabelled MAb were injected into the hepatic artery. The first 3 patients received F(ab')2 and the 4 last ones intact MAb. None of the 7 patients showed any significant side effect for a period of observation of 6–21 months, except for the last patient, who received two doses of 100 mCi of intact I-131 MAbs, and presented a severe leuco- and thrombocytopenia which was easily controlled by injection of allogeneic platelets. We observed good localisation of intact I-131 MAb in liver metastases, documented by ECT, and tumour radiation doses of about 2,500 rads per injection, but we have not yet obtained definite evidence of tumour regression. The last 3 patients had liver metastases of less than 80–120 g, which could be surgically resected 2 and 4 months after injection for two patients, or successfully treated by intra-arterial chemotherapy in the third patient. Two of these 3 patients have now a normal CEA and no evidence of disease, 17 and 21 months after injection.

### Prospects for neutron capture therapy

K.R. Durrant & C.B.T. Adams

*Department of Radiotherapy and Oncology, and Neurological Surgery, Churchill Hospital and Radcliffe Infirmary, Oxford, UK.*

Irradiation with low-energy neutrons of tissues containing high concentration of boron-10 leads to the neutron-capture reaction, with the production of very short-range densely ionising particles. The range of these particles is of the order of one cell diameter and there is little irradiation of non-borated cells.

This reaction occurs within a limited neutron energy range, and an intense neutron flux from a reactor is necessary to be of practical value. Similarly the intracellular boron concentration is critical.

Recent progress has been made in the selective boronation of tumour cells, and in the production of intense neutron energy fluxes of the optimum energy. For non-metastasising tumours of limited radio-sensitivity such as malignant gliomas, neutron-capture therapy offers the prospect of a major involvement in therapeutic results.

### Astatine-211 in cancer therapy

L.M. Cobb

*Department of Experimental Pathology and Therapeutics, MRC Radiobiology Unit, Harwell, Didcot, Oxon OX11 0RD, UK.*

The main emissions following the disintegration of astatine-211 are $\alpha$-particles of either 5.9 MeV or, from the $^{211}$Po daughter, 7.45 MeV. This radionuclide is of interest in targeted therapy because of the very significant cytotoxicity of $\alpha$-particles. Compared with the $\beta$-particles of iodine-131 or yttrium-90 they will produce approximately 200 times the number of DNA double strand breaks per nuclear traversal.

The isotope, which has a half-life of 7.2 h and is cyclotron-produced, is at present only used experimentally. When conjugated to a tumour-specific monoclonal antibody (MRC OX7) $^{211}$At produced 50% cure in mice bearing a T-cell lymphoblastic lymphoma (Harrison *et al.*, 1987, *NCI Monogr.*, **3**, 157). The therapeutic potential has also been demonstrated for astatine-211 when linked to 2-methyl-1,4-naphthoquinol diphosphate or methylene blue, both of which accumulate in certain tumours (Brown, I. *et al.*, 1986, *Appl. Radiat. Isot.*, **37**, 789).

There may be a place for $^{211}$At in thyroid therapy without the need for a targeting agent. Astatine is a halogen and, like iodine, is concentrated by the thyroid gland, where it can produce pathological changes (Cobb, L.M. *et al.*, 1988, *Human Toxicol.*, **7**, 529). When mice bearing xenografts of human thyroid tumour were injected with $^{211}$At as sodium astatide the radionuclide localised in significant amounts in both follicular and anaplastic carcinomas (Cobb L.M. *et al.*, 1989, *Radiotherap. and Oncol.*, **3**, 203), indicating its potential value in patients where iodine-131 is not expected to reach an effective concentration.

### Abstracts of members' proffered papers

### Increased incidence of breast cancer in first degree relatives of newly diagnosed breast cancer patients

S.A. Wallace[1], J.M. Birch[1], D. Teare[1], M. Harris[1], A. Howell[1] & R.A. Sellwood[2]

[1]*Departments of Epidemiology, Oncology and Pathology, Christie Hospital, Manchester, and* [2]*Surgery, Withington Hospital, Manchester, UK.*

There have been few unbiased, comprehensive surveys of breast cancer (BC) risk among relatives of BC patients and none from the UK. Therefore 432 consecutively diagnosed BC patients were interviewed to assess the incidence of BC in first degree relatives. Reported BCs were confirmed by histological review. 422 mothers, 544 sisters and 396 daughters were included in the analysis. Expected numbers of BCs, calculated from age specific incidence rates were compared with observed numbers. Overall there was a significant excess (relative risk (RR) 1.6; Poisson probability ($P$) $<0.001$) which was higher for sisters (RR 1.9, $P<0.01$) and daughters (RR 5.6, $P<0.01$) than mothers (RR 1.3, $P=0.2$). First degree relatives were stratified according to the following proband characteristics: age, menopausal status, laterality, histology and hormone receptor status. Expected numbers of BCs were calculated for each respective subgroup. Factors in the proband associated with high BC risk to relatives were: premenopausal status (RR 2.6, $P<0.001$), age $<50$ (RR 2.7, $P<0.001$), unilateral left BC (RR 2.1, $P<0.001$), ductal carcinoma (RR 1.7, $P<0.001$), and oestrogen receptor positive (ER+) (RR 1.7, $P<0.01$). Bilaterality in probands did not confer high BC risk to their relatives (RR 1.2). Highest risk was seen in sisters of patients age $<50$ (RR 5.4, $P<0.001$), daughters of patients with left BC (RR 8.9, $P<0.001$), daughters of patients with lobular carcinoma (RR 10.5, $P<0.03$) and daughters of patients with ER+ tumours (RR 8.5, $P<0.01$). We conclude that analysis of clinical characteristics in BC patients may be of use in selecting high risk women for screening from among their relatives and may indicate suitable subjects for laboratory investigations of genetic breast cancer.

### Essential fatty acids inhibit breast cancer

G.A. Pritchard, R.E. Mansel & L.E. Hughes

*Department of Surgery, University Hospital of Wales, Cardiff CF4 4XN, UK.*

High levels of dietary fat are implicated in the pathogenesis of human breast cancer. The type of fat as well as the quantity is important. We have studied the effect of supplementary dietary fatty acids on the growth of human MCF7 breast tumours in athymic mice. Results are shown in the table.

Primrose oil and fish oil are rich in polyunsaturated essential fatty acids (γ-linolenic acid 10%, eicosapentaenoic acid 28%) and inhibited tumour growth. Olive oil had no effect.

Several national groups in the USA have recommended halving dietary fat intake to reduce breast cancer risk. We would suggest that this be coupled with an increased balance of essential fatty acids.

| | | *Tumour weight (mg)* | | |
|---|---|---|---|---|
| *Diet* | *n* | *Median* | *Interquartile range* | *P (Mann–Whitney)* |
| Control | 24 | 271 | 149–453 | |
| 5% olive oil | 24 | 213 | 158–375 | 0.34 n.s. |
| 5% primrose oil | 24 | 133 | 37–297 | 0.009 |
| 5% fish oil | 24 | 70 | 19–205 | 0.001 |

### The effects of a low-fat diet on serum oestradiol and serum prolactin levels

I.L. Crighton[1], M. Dowsett[2], M. Hunter[3], C. Shaw[3] & I.E. Smith[1]

[1]*Department of Medicine,* [2]*Department of Biochemical Endocrinology, and* [3]*Department of Dietetics, Royal Marsden Hospital, Fulham Road, London SW3 6JJ, UK.*

There is a good epidemiological evidence associating *per capita* fat intake with the incidence of breast cancer worldwide (Wynder *et al.*, 1986, *Cancer*, **58,** 1804). Changes in fat intake may modify levels of circulating hormones (Rose *et al.*, 1987, *JNCI*, **78,** 623; Ingram *et al.*, 1987, *JNCI*, **79,** 1225), and thereby influence the risk of developing breast cancer.

For our study 18 pre- and 20 post-menopausal volunteers were recruited. After an initial dietary assessment and baseline blood sampling the volunteers were commenced on a low-fat diet (approx. 20% calories from fat). Blood samples were collected weekly for 4 weeks, and monthly thereafter for as long as the diet was continued. Fat content of the diet was assessed monthly by seven-day dietary records. The blood samples were assayed for serum prolactin (Prl) and oestradiol (E2) by radioimmunoassay. The results are shown below.

None of above differences are statistically significant.

| $x \pm$ *s.e.m. (n)* | *Base* | *After 4 weeks* |
|---|---|---|
| *Post-menopausal* | | |
| Prl (IU/l) | 109.5±12.3 (20) | 97.2±8.2 (20) |
| E2 (pmol/l) | 35.2±10.2 (20) | 25.9±5.9 (20) |
| *Premenopausal* | | |
| Prl (IU/l) | 152±15 (18) | 157±17.3 (18) |
| E2 (pmol/l) | 249±35.5 (14) | 434±106.2 (14) |

These preliminary studies have not demonstrated any significant changes in serum E2 and/or Prl levels as a result of a short-term low-fat diet. Further investigation into the long-term effects is in progress.

### Hormonal enhancement of the actions of cytotoxic drugs on breast cancer cells

N.A. Shaikh[1], A.M. Owen[2], M.W. Ghilchik[1] & H. Braunsberg[2]

[1]*Breast Clinic and* [2]*Department of Chemical Pathology, St Mary's Hospital and Medical School, London W2 1PG, UK.*

Clinical studies have suggested that pretreatment with oestrogen (cf. Harmsen & Porsius, 1988, *Eur. J. Cancer Clin. Oncol.*, **24,** 1099) or progestogen, possibly potentiated by oestrogen (Ghilchik *et al.*, 1987, *Br. Med. J.*, **295,** 1172) may enhance the beneficial effects of cytotoxic drug combinations. To investigate these claims and identify the drugs involved we have studied possible hormone/drug interactions in cultured human breast cancer cells. Using MCF-7 cells and a subline (MCF-7M) which does not respond to the growth-inhibitory action of medroxyprogesterone acetate (MPA), we have obtained convincing evidence that immediate pre-treatment with either oestradiol (E2), 1 nM 24 h, or MPA, 10 to 40 nM 48 h, can enhance the cytotoxic actions of 24 h exposure to methotrexate (MTX), vincristine (VCR) and adriamycin (ADR). The effects of MPA appear to be independent of its growth-inhibitory and glucocorticoid actions. Experiments conducted so far suggest that exposure to E2 before MPA treatment potentiates the effects of MPA on responses to MTX and ADR, but not VCR, while E2 alone under these conditions had no effect. The use of MPA, or sequential oestrogen and MPA, pretreatment in conjunction with cytotoxic drug offers much promise and deserves full clinical assessment.

### Evaluation of an mRNA selectively expressed in human breast cancer

Y.A. Luqmani, R. Skilton, R. McClelland & R.C. Coombes

*Clinical Oncology Unit, St George's Hospital Medical School, London SW17 0RE, UK.*

In an attempt to identify potential breast tumour markers, we have performed differential hybridisation with mRNA from normal and malignant breast tissue using a breast cancer cDNA library. One of the clones isolated, a 250 b.p. cDNA (Md2), was found to hybridise to a 6–700 nucleotide mRNA in 46% of 46 infiltrating ductal carcinomas. Expression was seen mainly in a subset of oestrogen receptor (ER) positive cancers but not in normal breast. Sequence data showed complete homology with the 3′ end of the pS2 gene (Jakowlew *et al.*, 1984, *Nucl. Acids Res.*, **12,** 2861).

In order to study its cellular localisation we used $^{35}S$-labelled Md2 for *in situ* hybridisation to frozen sections. We observed specific autoradiographic signals over the tumour cells of breast cancers with no significant reaction over stromal or vascular elements and only very rarely were grains seen over normal ductal epithelia above background.

To determine whether measurements of Md2 mRNA could be of value in predicting response to therapy we examined a group of 15 patients who had undergone primary endocrine treatment for recurrent disease. We found that expression of Md2 message in the primary tumour correlated well with the subsequent response of the patients to therapy ($P=0.02$). Its predictive value was the same as that provided by ER status: both measurements predicted outcome correctly in about 80% of this group. In view of its greater abundance, measurement of Md2/pS2 message could be a useful marker if these initial findings are consistent over a larger patient group.

**Development of a mouse monoclonal antibody against human urinary epithelial mucin for studies in breast cancer**

M.R. Price, J.A. Pugh, A. Clarke, M. Sekowski, C. O'Sullivan, E. Jacobs & J.F.R. Robertson

*Cancer Research Campaign Laboratories, University of Nottingham, and City Hospital, Nottingham, UK.*

Human urine is an abundant source of a high molecular weight polymorphic epithelial mucin, which is expressed by breast cancer and other carcinomas as well as on the luminal surfaces of normal glandular epithelia. The IgM anti-breast carcinoma monoclonal antibody, NCRC-11, reacts with this mucin, and an NCRC-11 antibody immunoadsorbent column was employed for its bulk purification directly from normal urine. The material isolated was used as the immunogen to produce 'second generation' monoclonal antibodies to complement the range of reagents reactive with breast carcinomas and available for further therapeutic and diagnostic applications. One antibody, C595 – an IgG3 antibody – was selected for this purpose. C595 reacted with mucins from breast and ovarian carcinomas, as well as with antigen from normal human urine and with material elevated in breast cancer patients' sera. The overall profile of reactivity of the new IgG monoclonal antibody, prepared against urinary mucin, was similar to that of the original IgM anti-breast carcinoma antibody, NCRC-11.

The strategy adopted in this investigation illustrates an approach for developing new customised antibody reagents with properties, such as class, subclass or fine specificity, which may be distinct from those of the 'parent' antibody.

**Chromosome 5 allele loss in familial and sporadic colorectal adenomas**

M. Rees[1], S.E.A. Leigh[1], J.R. Jass[2] & J.D.A. Delhanty[1]

[1]*Galton Laboratory, University College London, 4 Stephenson Way, London NW1 2HE, and* [2]*Department of Pathology, St Mark's Hospital, City Road, London EC1V 2PS, UK.*

DNA extracted from familial and sporadic colorectal neoplasms was compared with constitutional DNA using a range of hypervariable locus specific probes to assess the extent of allele loss during conversion to malignancy. Chromosome 5 allele loss was observed in 23% of carcinoma samples, as previously found by others (Solomon *et al.*, 1987, *Nature*, **328**, 616; Okamoto *et al.*, 1988, *Nature*, **331**, 273). However, we have been able to show for the first time loss of the D5S43 locus in adenomas from 20% of patients, the great majority of whom had the precancerous condition, adenomatous polyposis coli (APC). These results suggest significant genetic changes involving chromosome 5 are occurring in benign adenomas. Probes for chromosome 1 (loci D1S7 and D1S8) and for chromosome 7 (loci D7S21 and D7S22) revealed no notable alterations in the adenoma samples. Complete loss of alleles for loci on chromosome 7 was not observed in carcinomas but reduced intensity of one parental allele was found in 3 specimens one of which was known to have multiple copies of this chromosome. Results using probes for chromosome 1 suggest that deletion of the D1S7 or D1S8 loci is not a common event in colorectal carcinogenesis. Loss of chromosome 5 alleles in adenomas from APC patients provides evidence in support of Knudson's hypothesis.

**Homozygous deletions of 11p13 markers in Wilms' tumour DNAs**

R. Wadey, B. Buckle, J. Cowell & J. Pritchard

*ICRF Laboratory of Molecular Oncology, Institute of Child Health, Guildford St., London WC1N, UK.*

The association between aniridia and Wilms' tumour (WT) with constitutional heterozygous deletions of the short arm of chromosome 11 (11p) indicates the location of a WT predisposition gene (Wg). The shortest region of overlap for these deletions involves part of band 11p13. The isolation of the retinoblastoma (Rb) predisposition gene was facilitated by the presence of small homozygous deletions in a small percentage of Rb tumour DNAs. To identify any similar deletions in WT we used four recently isolated 11p13 sequences to probe primary WT DNAs. Two of these probes (p9RHD6.5 and D11S87) were found to be homozygously deleted in the same tumour (GOS 129). Neither of the other two probes (NBSC 12 and p56H2.4) were deleted or rearranged in any of the tumour DNAs tested.

In order to assign chromosome 11 markers to different regions within the 11p13 region we isolated three 11p deletions in somatic cell hybrids and characterised the position of the breakpoints using 11p markers including CAT and FSH$\beta$ (probes for the catalase and $\beta$FSH subunit, respectively). Using these hybrids three of the above probes (p9RHD6.5, D11S87 and NBSC 12) were mapped to the distal region of 11p13 between CAT and the aniridia locus (Ag). p56H2.4 was found to map to the proximal region of 11p13 between Ag and FSH$\beta$.

We are characterising GOS 129 DNA further, using flanking polymorphic 11p markers and pulse field gel electrophoresis and also constructing a Lambda library of GOS 129 DNA to try to isolate a clone spanning the deletion.

**Multiple mRNA species for GGT appear to be transcribed from a single gene**

S.A. Griffiths & M.M. Manson

*MRC Toxicology Unit, Woodmansterne Road, Carshalton, Surrey SM5 4EF, UK.*

Gamma glutamyl transpeptidase (GGT) is a plasma membrane-bound heterodimeric glycoprotein with tissue-specific expression during normal development and carcinogenesis. GGT is induced focally in rat liver by hepatocarcinogens and also periportally after treatment with the antioxidant ethoxyquin (EQ).

We have shown that the GGT mRNA induced in rat liver (2.4 kb) appears larger than the mRNA in normal rat kidney (2.2 kb). In order to study the expression of these two GGT mRNA species, we prepared a cDNA library in $\lambda$gt 10 from F344 rat liver treated with EQ (0.5% in the diet for 23 weeks). The library was screened using a probe containing two thirds of the Wistar rat kidney GGT cDNA (Laperche *et al.*, 1986, *PNAS*, **83**, 937). We isolated a clone of 2.2 kb which contained an internal *Eco* RI site and subcloned the two *Eco* RI fragments of 1.4 kb and 0.8 kb into M13 for sequencing.

Analysis revealed a sequence of 2,054 bp, plus 22 bases of the poly A tail, containing a reading frame of 1,704 bp, a 5′ untranslated sequence (UTS) of 203 bp and a 3′ UTS of 147 bp. The predicted amino acid sequence of induced rat liver GGT agrees with that of the rat kidney GGT with minor exceptions (Laperche & Guellaen, personal communication). Both sequences predict a protein of identical size. Southern blot data indicate a single gene for GGT, which, together with the divergence in the 5′ UTS of our liver GGT cDNA compared with the kidney cDNA, suggest differential splicing and the possibility of different promoters for tissue specific expression of GGT.

S.A.G. was supported by a grant from AICR. We thank J.A. Green for advice on computer analysis and P. Swann for synthesising oligonucleotides.

### Circulating lymphocyte subpopulations and NK cell activity in the rat model of azoxymethane induced colon neoplasia

K.M. Rigg, B.K. Shenton, T.W.J. Lennard & R.M.R. Taylor

*Department of Surgery, The Medical School, Newcastle upon Tyne NE2 4HH, UK.*

The azoxymethane induced colonic tumour in rats is a helpful model for the study of human colorectal cancer. We examined the effect of rat intestinal neoplasia on circulating lymphocyte subpopulations and natural killer (NK) cell activity. Twenty-four male Sprague–Dawley rats weighing 188 ± 21 g were injected with 15 mg kg$^{-1}$ azoxymethene s.c. weekly for six weeks. A control group of 24 weighing 187 ± 20 g were injected with saline. At 26 weeks the rats were anaesthetised in pairs and venesected. The azoxymethane group yielded 96 colonic and duodenal tumours. Lymphocytes were separated and stained with a panel of monoclonal antibodies for T-cells (OX19), T suppressor/cytotoxic (OX8), T helper (W3/25) and interleukin-2 receptor (OX39). Analysis was performed by flow cytometry. NK activity was determine by a 4 h $^{51}$Cr release cytotoxicity assay using labelled YAC-1 cells as targets at varying effector: target cell ratios.

| | *Lymphocyte phenotype* | | |
|---|---|---|---|
| *Subset* | *Control % median (range)* | *Tumour % median (range)* | *E:T ratio* |
| OX19 | 74 (57–88) | 67 (53–88)[a] | 100:1 |
| OX8 | 41 (20–47) | 40 (26–62) | 50:1 |
| W3/25 | 40 (23–48) | 39 (23–51) | 25:1 |
| OX9 | 8 (5–12) | 9 (6–16) | 12.5:1 |
| | *NK cell activity* | | |
| *Subset* | *Control % killing median (range)* | *Tumour % killing median (range)* | |
| OX19 | 53(11–99) | 32(7–64) | |
| OX8 | 43 (7–93) | 20(5–55) | |
| W3/25 | 38 (5–57) | 14(1–47)[b] | |
| OX9 | 30 (2–53) | 9(1–29)[b] | |

[a]$P<0.04$; [b]$P<0.01$, Mann-Whitney U test.

NK activity is reduced in the tumour bearing rat and is associated with fewer circulating T-cells. These immune defects associated with rat intestinal neoplasia provide a suitable model for the testing of specific immunomodulators.

### Nucleolar organiser regions (NORs) in dysplastic lesions and squamous cell carcinomas of the oral mucosa

S. Chungpanich & C.J. Smith

*Department of Oral Pathology, University of Sheffield, 31 Claremont Crescent, Sheffield S10 2TA, UK.*

A silver staining technique for NOR-associated proteins has been shown to distinguish benign from malignant lesions in various sites. This study aims to determine whether the technique predicts malignant potential in dysplastic lesions of oral mucosa by comparing AgNORs in groups of these lesions, in oral cancer and in normal mucosa. The original method of Ploton *et al.* (*Histochem. J.*, 1986, **18,** 5) has been modified by staining at 4°C. The material comprised 27 examples of oral epithelial dysplasia (OED), 10 oral squamous cell carcinomas (OSCC) and 10 controls of normal oral mucosa (NOM); all were formalin fixed and paraffin embedded. The OED specimens were subdivided into groups: 10 lesions coexisting with carcinoma (group A), 7 lesions with (group B) and 10 lesions without (group C) malignant development over the succeeding five years. The number of AgNORs per nucleus was counted under a × 100 lens separately in the basal and in the deepest three suprabasal layers (100 consecutive cells in randomly selected fields of each layer). In the basal layer, there were no statistically significant differences between mean AgNOR counts in OSCC (4.59 ± 0.93) and any group of OED (A = 4.35 ± 0.63, B = 5.26 ± 1.09, C = 5.30 ± 1.63), nor between the OED groups themselves. However, all had significantly higher counts ($P<0.05$) than NOM (3.45 ± 0.89). In the suprabasal layers, the mean AgNOR counts in groups B (6.35 ± 1.19) and C (6.63 ± 1.94) were significantly higher ($P<0.05$) than in OSCC (4.98 ± 1.22) and NOM (4.59 ± 0.82); those in group A (5.22 ± 1.94) were similar to carcinoma. Again there were no statistically significant differences between the three OED groups. The mean AgNOR counts do not appear to be reliable indices for assessing malignant potential of OED.

### Expression of p62 c-*myc* in the prognosis of colorectal cancer

S. Rowley, J.P. Neoptolemus, I.A. Donovan & M.R.B. Keighley

*Department of Surgery, Dudley Road Hospital and University of Birmingham, UK.*

Expression of the c-*myc* oncogene has been shown to be of some significance in the prognosis of carcinoma of the breast and cervix.

We have quantified the c-*myc* protein product (p62 c-*myc*) in colorectal cancer using archival paraffin embedded material. A double labelling technique was used on extracted nuclei with a mouse monoclonal antibody to p62 c-*myc* (MYC-1 6E10) and a second fluoroscein labelled rabbit antimouse immunoglobulin. DNA in the nuclei was counterstained with propidium iodide. The levels of p62 c-*myc* were quantified using a flow cytometer and expressed as fluorescence units (FU).

p62 c-*myc* was compared to stage and outcome in 47 patients. There was no relationship to Dukes stage: stage A ($n=6$, mean 13.5 FU, range 0–45); stage B ($n=21$, 44.8, 0–150); stage C ($n=13$, 60, 13–201); stage D ($n=7$, 32, 3–68).

Levels of p62 c-*myc* less than 20 FU were associated with significantly improved survival over 5 years (log rank analysis $\chi^2=4.76$, d.f.1, $P<0.05$).

These results suggest that p62 c-*myc* expression may be a prognostic index for colorectal cancer.

### Frequency of serum tumour marker monitoring in patients with non-seminomatous germ cell tumours

M.J. Seckl, G.J.S. Rustin & K.D. Bagshawe

*Department of Medical Oncology, Charing Cross Hospital, London W6 8RF, UK.*

In patients relapsing on surveillance following orchidectomy for stage I non seminomatous germ cell tumours, it is essential that treatment is initiated before they develop advanced disease with a poor prognosis. Patients who start chemotherapy with levels of human chorionic gonadotrophin (HCG) $>1,000\,\mathrm{i.u.\,l^{-1}}$ and/or alpha-fetoprotein (AFP) level $>500\,\mathrm{ku\,l^{-1}}$ have been shown to have a worse prognosis than patients with lower marker levels (*Lancet*, 1985, **ii**, 8). We studied 64 patients between 1968 and 1987 with rising serial tumour markers in an attempt to predict the time taken for HCG or AFP to rise to poor prognostic levels. Rates of marker increase could have resulted in adverse levels in 2 patients (3.1%) within 14 days, in 8 patients (12.5%) within 28 days and in 16 patients (25%) within 6 weeks. This suggests that initially, weekly marker estimations should be performed on stage I surveillance patients. The extra cost to a specialist follow-up laboratory of weekly as opposed to the usual monthly marker measurements will be less than £33,600 for every 400 patients on surveillance. One extra patient is likely to be cured for this sum.

**Tumour-associated trypsin inhibitor (TATI) as a marker of ovarian cancer**

J. Fisken, R.C.F. Leonard & J.E. Roulston

*University Departments of Clinical Chemistry and Clinical Oncology, Royal Infirmary, Edinburgh EH3 9YW and Western General Hospital, Edinburgh EH4 2XU, UK.*

TATI is a 6kD peptide, first isolated from the urine of a patient with ovarian serous cystadenocarcinoma. Putative roles include tumour protection and, conversely, host defence against tumour invasion. Elevated TATI has been found in urine, serum and tissue extracts from patients with gynaecologic malignancy. Particularly high levels occur in serum and cyst fluid from patients with mucinous ovarian carcinomas, which tend to be poor or non-secretors of CA125. A role of TATI in the management of ovarian mucinous cancer has therefore been suggested (H. Halila *et al.*, *Br. J. Cancer*, 1988, **57**, 304).

We assayed serum CA125 and TATI in 359 serial samples from 142 ovarian epithelial cancer patients. Of those currently assessed, 105 patients were followed up for 1–44 months (median 11 months). Overall sensitivity and specificity for TATI were 53% and 84% respectively, greater values than those obtained for PLAP (placental-like alkaline phosphatase) in the same patient cohort (J. Fisken *et al.*, *J. Clin. Pathol.*, in the press). Sensitivity was 9%, 25%, 54% and 73%, specificity was 88%, 88%, 80% and 100% for FIGO disease stages I, II, III and IV respectively. TATI assay detected 1 true positive (TP) result in 3 stage III patients where CA125 was false negative (FN), while 3 stage IV patients had consistently TP TATI levels ($>3\times$upper limit of normal) when CA125 was FN (1 mucinous, 1 endometroid and 1 serous papillary adenocarcinoma). TATI was positive in a further 4 patients, with negative CA125 levels, needing further clinical evaluation. Our preliminary analyses indicate that TATI may provide useful additional information in management of certain patients with ovarian cancer.

**Potent inhibition of oesophageal metabolism of methylbenzylnitrosamine, an oesophageal carcinogen, by higher alcohols present in alcoholic beverages**

V.M. Craddock

*MRC Toxicology Unit, Woodmansterne Road, Carshalton, Surrey SM5 4EF, UK.*

Consumption of alcoholic beverages is associated with oesophageal cancer, but ethanol *per se* has not been shown to be carcinogenic in animal experiments. Certain nitrosamines to which man is exposed are the most potent oesophageal carcinogens known. The concept that the action of alcohol is mediated through an effect on nitrosamine metabolism has been studied previously, but work was done mainly with nitrosamines not especially carcinogenic for oesophagus, and with liver microsomes, although each tissue has its distinctive spectrum of cytochrome P450 isozymes. Ethanol has been the alcohol studied, but cancer risk depends not only on the overall ethanol intake, but also on the type of alcoholic beverage. Apple brandy presents an exceptionally high risk, followed by other spirits, wine and beer. Apple-based alcoholic drinks contain unusually high levels of higher alcohols, grape brandies and whisky contain less, with much lower concentrations in wine and beer. Cancer risk is proportional to intake of higher alcohols.

The effect of ethanol and a major higher alcohol, 2-methylbutanol (2Mb), on metabolism of methylbenzylnitrosamine (MBzN), a potent oesophageal carcinogen, was studied using oesophageal and liver microsomes. 2Mb was approx. 1,000 times more potent an inhibitor than was ethanol.

The significance of the competitive inhibition could be its implication that the 2Mb is metabolised much more rapidly than is ethanol, presumably to form toxic aldehydes. 2Mb but not ethanol increased cell replication in the oesophagus. As with neonatal animals, increased vulnerability to nitrosamines is likely to be due to the higher rate of DNA replication, and to occur in spite of reduced nitrosamine metabolism, not because of it.

**The use of sustained release leuprorelin in advanced carcinoma of the prostate**

R.T.D. Oliver, A.J. Neal, M. Caulfield & S. Heard

*Department of Medical Oncology, The London Hospital, London, UK.*

Leuprorelin acetate (Leuprolide, Lederle Laboratories) is a synthetic gonadotrophin releasing hormone analogue. Twenty consecutive referrals with previously untreated metastatic carcinoma of the prostate, aged 47–80 years, were recruited into a non-randomised phase II trial between February 1987 and February 1988, to assess the efficacy and toxicity profile of this drug in a sustained release preparation. Patients received a monthly subcutaneous injection of 3.75 mg in 2 ml of diluent via a 23 gauge needle until disease progression. To date, 12/20 remain on Leuprolide, 4/20 have died and 4/20 have been withdrawn due to disease progression (median time on study for all patients = 10 months. One of 20 was castrate on starting treatment, and the remaining 19/20 attained castrate levels of testosterone (13 within 4 weeks). Nineteen of 20 had an elevated prostatic acid phosphatase, 14 attaining normal values. Fourteen of 20 had an abnormal isotope bone scan, and of these 7 attained a partial response. Eight of 20 had extraskeletal metastases, with 1 complete and 6 partial responses. Overall, 14/20 attained a partial response (NPCP criteria) at some point in the study. There were no serious injection site reactions, and no haematological or biochemical disturbances of note. We conclude that sustained release Leuprolide is a safe, effective and convenient alternative to agents currently available in the treatment of advanced carcinoma of the prostate.

**Preliminary study of aromatase inhibitor CGS16949A in advanced post-menopausal breast cancer**

R.C. Stein[1], M. Dowsett[2], J. Davenport[1], A. Hedley[1] & R.C. Coombes[1]

[1]*Clinical Oncology Unit, St George's Hospital Medical School, London SW17 0RE, and* [1]*Endocrine Department, Chelsea Hospital for Women, London SW3 6LT, UK.*

CGS16949A, a potent non-steroidal competitive aromatase inhibitor, has been used to treat 31 post-menopausal women with locally advanced or metastatic breast cancer. Doses of 0.3 mg BD, 1 mg BD and 2 mg BD orally were used. The median age of patients was 70 (range 51–90). Sixteen patients had ER positive carcinomas; the ER status of 12 was unknown. Nine patients (out of 17 who were assessed) had responded to previous endocrine therapy.

Twenty-eight patients, treated with CGS16949A for a median of 4 months (maximum 8) have been assessed for response by UICC criteria. Five patients all treated with 1 mg BD have responded and disease has stabilised in 15 patients. Disease sites in responding patients were soft tissue (4), bone (1).

Side effects were reported by 10 patients. One patient treated with 0.3 mg BD developed a vasculitic rash necessitating withdrawal from treatment. Two patients treated with 2 mg BD had troublesome nausea. Other side effects were mild and transient and included flushing, headache, giddiness, lethargy and disturbance of taste.

Oestradiol (E2) and oestrone level measured in 11 patients treated with both 0.3 mg BD and 1 mg BD were significantly suppressed after 2 weeks at both dose levels: E2, pre: $27.0 \pm 4.0$, 0.3 mg: $16.2 \pm 3.4$, 1 mg: $9.9 \pm 1.7$ pmol l$^{-1}$ ($x \pm$ s.e.m.). Serum aldosterone levels were significantly ($P < 0.02$) reduced at 1 mg BD: pre: $278 \pm 30$, 0.3 mg: $249 \pm 18$: 1 mg: $198 \pm 24$ pmol l$^{-1}$, $n = 10$. No change in gonadotrophins, SHBG and other adrenal hormones occurred. Endocrine data for 2 mg BD are not yet available.

Conclusions: CGS16949A is a clinically effective aromatase inhibitor. Endocrine data indicate that 1 mg BD is a more effective dose than 0.3 mg BD. Non-specific adrenal suppression may be a problem at higher doses.

### 4-Hydroxyandrostenedione alone and in combination with Zoladex in premenopausal breast cancer

R.C. Stein[1], M. Dowsett[2], A. Hedley[1] & R.C. Coombes[1]

[1]*Clinical Oncology Unit, St George's Hospital, London SW17 0RE, and* [2]*Chelsea Hospital for Women, London SW3 6LT, UK.*

The LHRH analogue Zoladex (ICI Pharmaceuticals) induces suppression of gonadotrophin dependent ovarian oestrogen synthesis and is effective treatment, but only for a limited period, in premenopausal breast cancer. Peripheral aromatasation is important in premenopausal women. We have therefore investigated the endocrine and clinical effects of the aromatase inhibitor 4-hydroxyandrostenedione (4OHA) (which is an effective treatment for advanced post-menopausal breast cancer) on its own and in combination with Zoladex in premenopausal women with advanced breast cancer.

Six women were treated with the maximum tolerated dose of 4OHA (500 mg i.m. weekly) for between 2 and 19 weeks. No significant reduction in oestradiol (E2) level occurred in any patient and no patient responded to treatment (NC: 1, PD: 5). Three (of 4) patients subsequently responded to Zoladex treatment.

The endocrine effects of the *combination* of 4OHA 500 mg i.m. weekly and Zoladex were studied in 5 patients who were maintained in remission with Zoladex treatment. Significant ($P < 0.01$) further suppression of E2 levels occurred 7 days after 4OHA treatment (from a mean of $23.6 \pm 4.1$ to $6.1 \pm 0.9$ pmol l$^{-1}$) but gonadotrophins did not change. The clinical value of combined treatment has been demonstrated by 1 (of 3) patient who responded, having previously experienced a remission and subsequently relapsed on treatment with Zoladex alone.

We conclude that 4OHA alone is not an effective treatment for premenopausal breast cancer. However, the combination of 4OHA and Zoladex produces more profound oestrogen suppression and may be a more effective treatment than Zoladex alone.

### The effect of medroxyprogesterone acetate on aromatase and DNA polymerase α activities in breast tumours

A. Purohit[1], M.J. Reed[1], A. Singh[1], N.G. Coldham[1], M.W. Ghilchik[2] & V.H.T. James[1]

[1]*Department of Chemical Pathology and* [2]*The Breast Clinic, St Mary's Hospital Medical School, London W2 1PG, UK.*

Progestogens such as medroxyprogesterone acetate (MPA) have been successfully used for the treatment of breast cancer. As it has been reported that progestogens can stimulate aromatase activity in endometrial tissue (Tseng *et al.*, *Endocrinology*, 1986, **118**, 1312), we have examined the effect of treatment with MPA on tumour aromatase activity as measured using an *in vitro* technique. The activity of DNA polymerase α, which provides an index of cellular proliferative activity, and plasma levels of cortisol, androstenedione and oestrone were also measured. Blood and tumour samples were obtained before and after treatment with MPA (500 mg day$^{-1}$, i.m., for two weeks). Aromatase activity increased significantly in 4/6 specimens examined whereas DNA polymerase α activity decreased in all samples by $57 \pm 25\%$ (mean $\pm$ s.d.). Plasma cortisol, androstenedione and oestrone concentrations were significantly reduced after treatment with MPA. It is concluded that although treatment with MPA appears to increase aromatase activity in some tumours this is associated with a decrease in DNA polymerase α activity. This suggests that the amount of oestrogen formed in tumours after treatment with MPA is reduced. The reduction in plasma concentration androstenedione that results from the adrenocortical suppression associated with the use of MPA, suggests that less substrate will be available for conversion to oestrogen, in spite of the increase in aromatase activity found in some tumours after treatment with MPA.

### Circulating CA 15-3 in patients with early breast cancer

H.G. Kynaston[1], R. Fifield[2], R.E. Mansel[1] & D.J.T. Webster[1]

[1]*Department of Surgery, University Hospital of Wales, Heath, Cardiff, and* [2]*Protein Laboratory, Cardiff Royal Infirmary, Newport Road, Cardiff, UK.*

CA 15-3 is a recently described breast cancer associated antigen, defined by two different monoclonal antibodies; DF3, raised against a membrane enriched fraction of human breast cancer cell line and 115 D8, raised against antigens of human milk fat globule membranes. It has been claimed that these breast associated antigenitic determinants CA 15-3 have been detected at elevated levels in the sera of breast cancer patients but not elevated in the sera of the majority of normal individuals (Hayes *et al.*, 1986, *J. Clin. Oncol.*, **4**, 1542).

In order to establish its value in the diagnosis of early breast cancer and its possible use as an adjunct to breast cancer screening a study was undertaken to evaluate CA 15-3 levels in patients admitted for a diagnostic excision biopsy of a possible cancer and patients with early breast cancer. Of 47 patients with early breast cancer, mainly stage 1 disease, only 5 patients (10.6%) had a CA 15-3 level greater than the upper limit of normal of 30 units per ml. Those patients with micronodal metastases to regional axillary lymph nodes, examined following mastectomy, had a similar distribution of serum CA 15-3 levels to those patients without micronodal involvement. Of 50 patients who underwent excision biopsy of a breast lump which proved to be benign 4 patients (8%) had an elevated CA 15-3 level of greater than 30 units per ml.

Serum CA 15-3 estimations have not been found to be useful in the investigation of patients with possible early breast cancer due to its low sensitivity and therefore cannot be recommended as an adjunct to mammography in breast cancer screening.

### Glycoprotein expression in normal and malignant breast cell lines

Z. Rayter[1] & R.A.J. McIllhinney[2]

[1]*Department of Surgery, St Peter's Hospital, Guildford Road, Chertsey, Surrey, UK, and* [2]*MRC Unit of Anatomical Neuropharmacology, University Department of Pharmacology, South Parks Road, Oxford, UK.*

Glycoprotein expression in normal human breast epithelial cells has been compared with that in 5 *bona fide* breast cancer cell lines MCF-7, T47-D, ZR-75-1, PMC-42 and Ca2-10, with two SV40-transformed breast cell lines SVE-3 and H-7, and with HBL 100 and MDA-MB-231. Two-dimensional gel electrophoresis has been employed to separate detergent-extracted glycoproteins which have been identified using a $^{125}$I-Con A overlay. The five *bona fide* breast cancer cell lines displayed similar electrophoretic patterns and intermediate homology (60–71%) with normal breast cells, although their patterns were somewhat simpler. All of these cell lines expressed two common novel glycoproteins, and each cell line expressed other novel glycoproteins idiosyncratically. SVE-3 and H-7 showed 77% homology with normal breast and also expressed one of these glycoproteins. HBL100 and MDA-MB-231 displayed 47 and 51% homology with normal breast cells, implying a poorly differentiated or non-epithelial cell of origin, consistent with their lack of differentiated breast cell function (Engel, L.W. & Young, N.A., 1978, *Cancer Res.*, **38**, 4327). We conclude that although breast cancer cells lose Con A-acceptor glycoproteins, they also express novel glycoproteins, two of which are common to all the cell lines, and some of which are expressed idiosyncratically in other transformed breast cell lines.

### DNA index S-phase fraction and prognosis in breast cancer

S.M. O'Reilly, R.S. Camplejohn, D.M. Barnes, R.R. Millis & M.A. Richards

*ICRF Clinical Oncology Unit, Guy's Hospital, London SE1 9RT, UK.*

DNA index and S-phase fraction (SPF) were measured by flow cytometry on paraffin embedded tissue from 140 primary breast tumours. All patients has stage I or stage II breast cancer diagnosed between 1973 and 1980, and the group was selected so that 50% had developed recurrent disease by the time of this study. The results of DNA analysis were compared with the size, degree of axillary node involvement, histological grade and steroid receptor content of the tumours, as well as with the patients' subsequent clinical course.

Forty-four of the 140 (31.4%) were diploid. SPF was evaluable for 134 (95.7%). The median SPF of the whole population was 7.1% with diploid tumours having a significantly lower SPF (3.2%) than aneuploid (10.1%, $P<0.001$). Both aneuploidy ($P=0.002$) and high SPF ($P<0.0001$) were strongly associated with high histopathological grade. There was no significant association between DNA ploidy or SPF and tumour size, nodal status or steroid receptor content. An SPF below the median was strongly associated with better relapse free survival ($P=0.008$), survival from diagnosis ($P=0.004$), and survival after relapse ($P=0.001$). Ploidy did not correlate significantly with clinical course.

Multivariate analysis showed that nodal status, tumour size and SPF were independent prognostic factors, but that the significance of SPF was lost when histological grade was included in the analysis.

### Alteration in the polyamine content of human breast cancer cells in response to stimulation and inhibition of cell growth

H.M. Wallace & S.J. Cross

*Departments of Medicine and Therapeutics, and Pharmacology, University of Aberdeen, Aberdeen, Scotland, UK.*

The polyamines are naturally occurring cellular polycations essential for optimal rates of cell growth and differentiation. Elevated concentrations of these molecules are present in many types of cancer cells where they are believed to be involved in the 'up regulation' of cell growth. The aim of this study was to determine the changes in intracellular polyamine concentrations in human breast cancer cells under conditions of increased (treated with 17$\beta$-oestradiol) and decreased (treated with methylglyoxal bis guanylhydrazone; MGBG) cell growth. ZR-75-1 cells were grown routinely in Dulbecco's medium supplemented with 10% (v/v) horse serum at 37°C in a humidified atmosphere of 95% air/ 5% $CO_2$. Cell growth was measured as the change in protein content (Lowry *et al.*, 1951, *J. Biol. Chem.*, **193**, 265) and polyamine content was measured by hplc (Wallace *et al.*, 1988, *Biochem. J.*, **253**, 223). Growth of ZR-75 cells was increased by treatment with 17$\beta$-oestradiol. This increase was accompanied by a similar increase in the total polyamine content in particular an increase in intracellular spermine concentration. MGBG, as inhibitor of polyamine biosynthesis, inhibited growth in a dose-dependent manner. The decrease in growth rate was paralleled by a decrease in spermine content. A combination of MGBG and 17$\beta$-oestradiol resulted in a partial recovery of cell growth and spermine content. Therefore it may be that spermine is the polyamine involved in the regulation of growth of these breast cancer cells.

### Characterisation of new hormone-response transplantable rat mammary carcinomas

S.A. Eccles & H.P. Purvies

*Section of Medicine, Institute of Cancer Research, Sutton, Surrey SM2 5NG, UK.*

The preclinical evaluation of new anti-endocrine agents can be aided by the use of appropriate hormone-sensitive tumour systems. Models commonly employed include primary chemically induced rat tumours, and human mammary tumours grown as xenografts in nude mice. In order to overcome some of the difficulties associated with such systems, we set out to develop transplantable hormone-responsive tumours with reproducible growth and oestrogen sensitivity in syngeneic rats.

We now describe the characteristics of 2 groups of such tumours: The HOSP series arose spontaneously in aged CBH/Cbi rats, OES lines were developed from a tumour induced by implantation of an oestrogen pellet. Both types of tumour are hormone-responsive in that their growth is enhanced by oestrogen supplementation and totally inhibited in males or ovariectomised females. In addition, tumours regress or show stasis of growth in response to treatment with 4-hydroxyandrostenedione, aminoglutethimide and Tamoxifen. Under certain dosage schedules Tamoxifen was shown to be 'oestrogenic' and, like oestrone, enhanced both haematogenous and lymphatic metastasis.

These cell lines are now being used to screen new anti-endocrine drugs, and to investigate autocrine and paracrine influences in the progression of mammary carcinomas towards hormonal independence and metastasis.

**Erythrocyte membrane stearic:oleic acid ratios in breast and colorectal cancer**

J.P. Neoptolemos[1], B.S. Thomas[2] & I.S. Fentiman[3]

[1]*University Department of Surgery, Dudley Road Hospital, Birmingham B18 7QH,* [2]*ICRF, Lincoln's Inn Fields, London WC2A 3PX, and* [3]*ICRF, Clinical Oncology Unit, Guy's Hospital, London SE1 9RT, UK.*

A previous report (Wood *et al*., 1985, *Br. Med. J*., **291**, 163) had shown increased oleic acid in erythrocytes associated with solid malignancies and the stearic:oleic acid ratio was proposed as a sensitive diagnostic test. This could not be substantiated (Soreide *et al*., 1987, *Br. Med. J*., **294**, 548) but there were difficulties in the methodology of both studies.

Samples obtained from two units specialising in breast and colorectal cancer were analysed 'blind' for the erythrocyte membrane stearic:oleic acid ratios using appropriate techniques. Fatty acids were extracted from membranes following saponification and methylated. Analysis was performed by gas liquid chromatography using a CPSil 88 fused silica quartz capillary column (50 m x 0.32 mm) with flame ionisation detection. A total of 167 subjects were studied. The mean stearic:oleic acid ratio ($\pm$s.d.) in 44 breast cancer patients, 1.24 (0.26) was not significantly different from 30 healthy controls, 1.10 (0.31) or 18 benign breast disease cases, 1.20 (0.37). Similar results were obtained from 34 colorectal cancer cases, 1.08 (0.29) and their 41 hospital controls, 1.09 (0.32). No relationship was found between age, sex, grade of tumour differentiation or stage of cancer and this ratio. The erythrocyte stearic:oleic acid is of no diagnostic value in patients with breast or colorectal cancer.

**Razoxane adjuvant chemotherapy in Dukes' C colorectal cancer**

K. Hellmann[1], J.M. Gilbert[2], J.T.C. Irwin[3], M. Evans[1] & P.G. Cassell[2]

[1]*The Westminster Hospital, London SW1,* [2]*Wexham Park Hospital, Slough, Berkshire, and* [3]*ICRF Cancer Chemotherapy Unit, Westminster Hospital, London, UK.*

Adjuvant treatment for resectable colorectal cancer has so far failed to show any effect. Razoxane (Rz) was therefore used because it had previously been shown to be effective as a single agent in CRC. One hundred and three Dukes' B and 89 Dukes' C patients were randomised to receive either usual clinical care (UCC) or UCC with Rz (p.o. 125 mg b.d., 5 days per week).

At 5 years, recurrence free interval (RFI) and survival were not significantly different for Dukes' B patients. For Dukes' C patients, on the other hand, RFI was significantly longer in Rz treated patients (by log rank analysis $P<0.05$). Differences in survival were just outside statistical significance. Some patients, however, although randomised to Rz, did not take any. When analysed according to whether or not patients received Rz, Dukes' C patients taking Rz did show a significantly longer survival (by log rank $P<0.05$). Toxicity was minimal, but 3 patients on Rz developed acute leukaemia after 1.5, 3 and 6 years of continuous Rz treatment. Therefore treatment now ceases at 2 years for Dukes' B and 3 years for Dukes' C. At 2 years the RFI for the 'as treated' groups in Dukes' B is 76.7% for Rz treated and 67.3% for controls ($P>0.1$), and 80.8% against 74.0% for survival ($P>0.1$). For Dukes' C patients at 3 years RFI is 41.8% in patients receiving Rz compared with 24.7% in the untreated ($P<0.025$) and 40.1% of Rz treated patients are alive compared with 25.5% of the untreated patients ($P<0.05$).

This was the first time that adjuvant chemotherapy showed any significant benefit on the Dukes' C group of colorectal cancer patients. These results warrant further examination of adjuvant Rz in Dukes' C colorectal cancer.

**Potential of Fab/c fragment for cancer therapy**

S. Demignot, M.C. Garnett & R.W. Baldwin

*Cancer Research Campaign Laboratories, University of Nottingham, Nottingham NG7 2RD, UK.*

$F(ab)_2$ and Fab fragments have shown no advantage over whole antibody for tumour therapy mainly because the improved diffusion is offset by a more rapid clearance from the body. Arend & Silverblatt (1975, *Clin. Exp. Immunol*., **22**, 502) showed that the Fc portion seems to control the catabolism of the antibody. We have therefore investigated the Fab/c fragment, which is the same size as $F(ab)_2$ but contains an Fc portion. If Fab/c clears at the same rate as antibody and maintains a good affinity for the target antigen, it should concentrate better in the tumour. Fab/c fragment was obtained by DEAE ion exchange and gel-permeation chromatography purification of pepsin digested mouse monoclonal antibody IgG2b 791T/36.

Blood clearance curves of iodinated whole antibody and Fab/c fragments were similar in shape, but Fab/c fragment curves were always below the Ab curve. This suggests that the Fab/c fragment diffused better but was catabolised more rapidly than antibody. Biodistribution experiments showed that Fab/c fragments diffuse significantly more rapidly throughout the body; the carcase in particular. During the catabolic phase, for the same blood concentration of Ab and Fab/c fragment there would be 25% more Fab/c fragment than Ab in the carcase. Expressed as % of injected dose, there was more Fab/c in the carcase during the distribution phase (17 hours after injection) and equal amounts when the equilibrium is reached, confirming the slightly enhanced catabolic rate of Fab/c fragment compared to whole antibody.

**Tumour associated antigen expression in colorectal cancers and their metastases**

A.J. Jewkes, M.C. Crowson, F. Macdonald & J. Crocker

*Surgical Immunology Unit, Department of Surgery, Queen Elizabeth Hospital, Birmingham B15 2TH, UK.*

Heterogeneity of tumour associated antigen expression (TAA) between colorectal primary tumours and their metastases have seldom been investigated. Previous histochemical studies have concentrated on primary tumour antigen expression and few metastatic lesions have been examined. Histological sections from 20 colorectal primaries and 74 synchronous lymph node metastases were examined by indirect immunoperoxide staining. Seven monoclonal antibodies to TAAs were used: 4 anti-CEA and 3 non-CEA antibodies (CA19-9, HMFG1, HMFG2). On two separate occasions each section of tumour was graded independently, by two observers, for tumour differentiation, the number of tumours positively stained by each antibody, the degree each antigen was expressed by each tumour, and the difference in cell expression between different antibodies in the same tumour.

All primary tumours expressed CEA to some extent but none were shown to have 100% expression of any single epitope. None of the non-CEA antigens were universally expressed by the primary lesions. The degree of expression of CEA and non-CEA antigens was not related to tumour differentiation. CEA negative tumours only rarely express other TAA used in this study. Heterogeneity of TAA expression was demonstrated between primary tumours and their metastases and between metastases from the same tumour with all antibodies studied. Metastases negative for CEA rarely expressed any of the non-CEA antigens. To accurately stage and treat colorectal cancer using radiolabelled MAbs, it is essential that all tumour sites express the appropriate TAA. This study clearly shows this is not the case. Investigation of agents to increase TAA expression might prove useful.

**Augmentation of CEA expression in gastrointestinal cancer**

A.J. Jewkes, F. Macdonald, B. Russell, C. Jones, D. Croom & W.H. Allum

*Surgical Immunology Unit, Department of Surgery, Queen Elizabeth Hospital, Birmingham B15 2TH, UK.*

Heterogeneity of CEA expression is common to most human gastrointestinal cancers and has important implications for the use of monoclonal antibodies in diagnosis or therapy of these tumours. Agents which could render CEA expression more homogeneous could result in improved antibody binding and more efficient diagnostic and therapeutic results. The level of expression of certain tumour associated antigens has been reported to be altered by recombinant interferons and theophylline.

We have investigated the effects of these agents on CEA expression in 4 colorectal cancer cell lines (LS174T, Colo 320, HT-29, LoVo), a gastric cancer cell line (MKN45) and human fibroblasts. All cell lines have been well characterised and cover a wide range of natural CEA expression. The drugs were added to cell culture in varying concentrations and incubated for 24 hours. Utilising flow cytometry with a specific murine monoclonal anti-CEA antibody (11-285-14), we found CEA expression was significantly increased ($P<0.001$) in certain cell lines with $\alpha$ interferon (Colo 320, LoVo) and with theophylline (HT-29, Colo 320, LoVo). Augmentation of CEA expression appeared dose dependent. Two cell lines were resistant to the effects of both drugs (LS174T, MKN45). Fibroblasts could not be induced to express CEA. Response was not related to tumour cell differentiation or initial level of CEA expression. Cell surface receptors are necessary for these drugs to alter antigen expression and heterogeneity of receptor expression could account for the variable response of the cancer cells studied. Although both agents can substantially increase CEA expression in some gastrointestinal cancers, the effect is not universal and may limit its effectiveness in the diagnosis and treatment of human cancer with antibodies.

**Repeated antitumour antibody therapy in man: use of cyclosporin A to suppress the host response**

J.A. Ledermann, R.H.J. Begent, A.M.B. Kelly, S.R. Wood, D.B. Smith & K.D. Bagshawe

*Cancer Research Campaign Laboratories, Department of Medical Oncology, Charing Cross, and Westminster Medical School, London W6 8RF, UK.*

Therapy of cancer with intravenous antitumour mouse monoclonal antibodies alone or conjugated to radionuclides or toxins has produced tumour responses but these are seldom sustained. Repeated therapy would probably be more effective but is prevented by the formation of human antimouse antibody (HAMA) after one or more injections of antitumour antibody. Cyclosporin A (CsA) has recently been shown to prevent the antibody response to repeated injections of mouse monoclonal antibodies in rabbits (Ledermann *et al.*, *Br. J. Cancer*, in the press). This study investigates the effect of CsA on the formation of HAMA in patients with CEA-producing tumours treated with repeated doses of a 131-iodine-labelled mouse monoclonal antibody to CEA. In 3 patients receiving no CsA the mean HAMA level had risen to 1,998 $\mu g\,ml^{-1}$ 2 weeks after the second injection of antibody. HAMA production was suppressed in 3 patients receiving high dose CsA (24 $mg\,kg^{-1}\,day^{-1}$ for 6 days starting 3 days before each antibody injection) and in 3 patients given low dose CsA (15 $mg\,kg^{-1}\,day^{-1}$ continuously starting 3 days before first antibody injection). HAMA levels were 3.5 $\mu g\,ml^{-1}$ and 1.97 $\mu g\,ml^{-1}$ 2 weeks after the second injection of antibody for high and low dose respectively. Up to four courses of antibody could be given to patients in whom HAMA was suppressed. Blood CsA levels were between 1,000–1,500 $ng\,ml^{-1}$ for the high dose group and 200–400 $ng\,ml^{-1}$ for the low dose. Nausea, vomiting and anorexia were severe and prolonged with high dose CsA but were less frequent and less severe with the low dose. WHO grade 1 renal toxicity was seen in both groups. Low dose CsA provides satisfactory suppression of HAMA with more acceptable side effects than a high dose CsA regimen. This allows the administration of repeated antibody injections and increases the potential for effective antibody targeted therapy of cancer.

**A preliminary study of the biodistribution of intraperitoneal 791T/36 antibody in ovarian cancer**

M.V. Pimm[1], A.C. Perkins[2], C. Gie[3], R.A. Marksman[1], L.D. Durrant[1], V.S. Byers[4] & R.W. Baldwin[1]

[1]*Cancer Research,* [3]*Obstetrics and Gynaecology,* [2]*Medical Physics, University of Nottingham, UK, and* [4]*Xoma Corp., California, USA.*

Monoclonal antibodies are potential vectors for targeting diagnostic isotopes or therapeutic agents to tumours. Ovarian cancer is largely confined to the peritoneal cavity and may be more effectively treated by intraperitoneal (i.p.) rather than systemic (i.v.) injection. In the present study, cells from ovarian tumours were shown to react with the 791T/36 antibody. Patients with stage III disease were given $^{131}I$ or $^{111}In$ labelled 791T/36 antibody i.p. Gamma camera imaging was performed immediately and at 48 hours, the latter before and after peritoneal lavage. Blood kinetics and urinary excretion were followed and radiolabel quantified in resected tumour specimens. The development of anti-mouse antibodies (AMA) was assessed by an ELISA assay.

Blood levels of both radiolabels rose over the first 20–40 hours reaching 8–14% of the dose in the total circulation and then declined with $T_{1/2}$ of 40 hours. Radiolabel was still attached predominantly to antibody as shown by precipitation with anti-mouse IgG antiserum. There was little urinary excretion of $^{111}In$ (<4% by 4 days) but $^{131}I$ was excreted ($T_{1/2}$ =42 hours). Interpretation of the images was less clear than in previous studies with antibody given i.v. (Powell *et al.*, 1987, *Am. J. Gynecol.*, **157**, 38) but in patients undergoing laparotomy there was agreement between site of uptake and surgical findings. Resected tumour specimens had up to 0.02% of the dose $g^{-1}$ but because radiolabel appeared in the blood it is unclear whether tumour uptake was from the circulation or local infiltration. All patients produced AMA.

Further studies are intended to determine the most appropriate route of administration of antibody or conjugates for therapy of ovarian cancer.

### *In vivo* distribution and fate of protein microspheres in the rat

N. Willmott[1], J.A. Goldberg[2], Y. Chen[1], C. McArdle[2] & A.T. Florence[1]

[1]*Department of Pharmacy, University of Strathclyde, Glasgow and* [2]*Department of Surgery, Royal Infirmary, Glasgow, UK.*

Protein microspheres were developed in order to (a) localise cancer therapeutic agents (cytotoxic drugs, radionuclides) in desired organs, (b) prolong residence time of these agents at the site of action, (c) biodegrade to permit multiple dosing. Our previous studies showed that microspheres (20–40 $\mu$m mean diameter) are trapped in target organ capillaries of animals and patients after injection at the appropriate point in the vascular system: diversion of microspheres to tumour deposits in target organs is possible with vasoactive agents by selectively constricting blood vessels supplying normal tissue. Once embolised a number of factors influence efficacy of incorporated therapeutic agents, e.g. residence time of microspheres at site of action which is determined by rate of biodegradation. In this study we have examined rate of biodegradation and subsequent fate of $^{125}$I-labelled microspheres prepared from albumin and casein following intrahepatic arterial injection (liver as target organ) and i.v. injection (lung as target organ). Rate of biodegradation is expressed as T-50 i.e. time take for loss of 50% of radioactivity initially localised in target organ. *In vitro* studies showed both types of microsphere to be stable in serum at 37°C. *In vivo* studies showed that in both liver (T-50 albumin = 3.6 days; T-50 casein = 6.8 days) and lung (T-50 albumin = 2.0 days; T-50 casein = 3.5 days) casein microspheres biodegraded more slowly that albumin.

The distinctive biodistributive characteristics and control over rate of biodegradation offered by this novel system may prove useful in delivery of cytotoxic drugs and radionuclides in cancer therapy and also help to elucidate the importance of duration of exposure to these agents.

### Childhood malignant liver tumours in the UK 1968–1984: the importance of complete surgical resection

E.A. Shafford[1], J. Pritchard[1], G.J. Draper[2], C.A. Stiller[2] & J.W. Keeling[3]

[1]*Hospitals for Sick Children, London,* [2]*CCRG, Oxford and* [3]*John Radcliffe Hospital, Oxford, UK.*

To study trends in survival and advances in chemotherapy and surgery for childhood malignant liver tumours, a survey was undertaken of 169 cases occuring in the UK from 1968 to 1984. There were 105 hepatoblastoma (Hbl) 33 hepatocellular carcinoma (HCCa) 26 other tumours and 5 whose histology is in doubt. The male/female ratio was 1:1.1 for Hbl and 1:1.09 for HCCa. Median age at diagnosis was 17 months and 9 years 11 months respectively. Commonest presenting symptoms for Hbl and HCCa were abdominal mass/distension 67%:50%, anorexia 37%:32%, and lethargy 29%:37%. Jaundice occurred in 18% HCCa but only 6% Hbl. 58% Hbl were anaemic at diagnosis compared to 33% HCCa. 36/56 (64%) Hbl and 10/24 (42%) HCCa had platelet counts $>500 \times 10^9 l^{-1}$. Serum AFP was elevated in 50/56 (89%) Hbl and 9/13 (69%) HCCa but was normal in 6 Hbl and 4 HCCa. Two Hbl have a family history of familial adenomatous polyposis. The majority of patients (63% Hbl and 76% HCCa) had stage III and IV disease at diagnosis. Three year survival for 1968–74 is 15% Hbl and 11% HCCa compared to 28% Hbl and 21% HCCa for 1975–84. 45 Hbl and 9 HCCa had complete surgical resection of primary tumour; 44% of each survive at 3 years. Only 1/60 Hbl and 0/23 HCCa survive after incomplete resection. There are no survivors of local relapse but 3/21 Hbl survive after metastatic recurrence. Thus complete surgical resection is of paramount importance in the survival of patients with hepatocellular tumours. The trend to improved survival over the two periods is probably due to improvements in chemotherapy and surgical techniques.

Supported by the Thomas Kately Fund and the South Essex Medical Education and Research Trust.

### High rate of complete surgical resection (CSR) in advanced hepatoblastoma (Hbl) after cisplatinum/doxorubicin ('PLADO') chemotherapy

E.A. Shafford[1], J. Pritchard[1], P.N. Plowman[1], V.A. Broadbent[1], L. Spitz[1], E.H. Howard[2], A.P. Mowat[2] & G. Mieli-Vergani[2]

[1]*Hospitals for Sick Children, London, and* [2]*Kings College Hospital, London, UK.*

Two-thirds of patients with Hbl present with advanced disease and the biggest obstacle to cure is conversion of unresectable to resectable tumour. Since 6/83 13 consecutively diagnosed patients with advanced Hbl have been treated with cisplatinum (PLA = 100 mg m$^{-2}$) and doxorubicin (DO = 50 mg m$^{-2}$ as bolus) = 'PLADO' 3 weekly, prior to and/or following surgery. The 8 males and 5 females were aged 1–53 months (med 17 months). Serum AFP at diagnosis ranged from 409–1,700,000 $\mu$g l$^{-1}$. Eight patients had stage IIIA tumours (macroscopic residual inoperable), 1 stage IIIB (multifocal primary) and 4 stage IV (lung metastases). One patient had a transient response to 'PLADO' but died 4 months from diagnosis, of progressive tumour. One patient had initial surgery followed by 'PLADO'. The other 11 patients showed sustained response to chemotherapy (CT) alone with a steep fall in serum AFP values, 2 patients having normal levels ($<5\,\mu$g l$^{-1}$) prior to surgery. Lung secondaries cleared in all 4 stage IV patients. Two patients have not yet come to surgery but the remaining 9 patients all had CSR of primary tumour at a median of 4.5 months (range 2–6 months) from diagnosis. One patient has since had a local relapse and died, a second patient has had a metastatic recurrence but is disease free after further treatment, the rest have completed therapy and survive 7–44 months from diagnosis. DO cardiomyopathy occurred in 2 patients (total doses 400 mg m$^{-2}$ and 350 mg m$^{-2}$). The first died in cardiac failure; the other survives, on digoxin, 39 months from diagnosis.

We conclude that 'PLADO' with surgery is effective treatment for advanced Hbl with substantially higher CSR than in our previous experience, without the use of alkylating agents and with a median duration of CT of only 5 months. Drug toxicity, especially DO, is of concern, but all survivors have excellent quality of life. Two teenage patients with multicentric hepatocellular carcinoma (HOC) had good clinical/AFP responses to 'PLADO': the combination therefore seems worthy of a trial in adults with HOC.

Supported by the Thomas Kately Fund and the ICRF.

### Importance of schedule for giving methotrexate and cisplatinum in combination to treat patients with transitional cell carcinoma

R.T.D. Oliver & S.J. Popert

*Department of Medical Oncology, The London Hospital, London, UK.*

As both metrotrexate and cisplatinum are nephrotic drugs their use in combination has always been associated with the risk of increased nephrotoxicity. As a consequence most regimens using the two drugs give methotrexate 24 h prior to cisplatinum. Following reports from Kaye showing that giving platinum at the same time as methotrexate did not alter methotrexate clearance, a phase 2 study was initiated giving both drugs synchronously. Fifty-one patients receiving this schedule, (group 1) and 53 patients receiving methotrexate 24 h prior to cisplatinum (group 2) have been studied. We found that 28 of 51 patients in the first group developed renal impairment sufficient to necessitate a reduction in dosage of cisplatinum or termination of treatment. This compared with 15 of 53 patients in the second group. ($P=0.01$). It is concluded that it is not wise to give methotrexate and cisplatinum without a 24-h gap in between.

**Comparison of cyclophosphamide, adriamycin, vincristine (CAV) and ifosfamide, etoposide (IE) in small cell lung carcinoma (SCLC): a randomised study**

S.W. Watkin, J.A. Green & H.M. Warenius

*CRC Department of Radiation Oncology, Clatterbridge Hospital, Wirral L63 4JY, UK.*

High response rates have been reported for several new drugs including etoposide, ifosfamide and cisplatin in the treatment of SCLC in various recent non-randomised studies. In the present study, 90 patients with SCLC have been randomised to receive either CAV (Greco, 1978, *B.M.J.*, **ii**, 10) or IE (I 5 $gm^{-2}$ i.v. day 1 with Mesna; E 100 $mgm^{-2}$ i.v. day 1–3) every 21 days, given to complete remission + 2 cycles. Patients in CR are randomised to receive thoracic irradiation (45 Gy in 15 fractions) or no further treatment. Fifty-nine patients have completed chemotherapy: 35 male, 24 female; median age 62 years, range 41–71 years. Median ECOG Performance status is 1 on both arms of the study and the mean number of cycles of chemotherapy is 4 on each arm. Reponse rates for regime A are CR 12 (42%), PR 7 (24%), SD 7 (24%), PD 3 (10%), NE 2 and regime B, CR 8 (33%), PR 7 (29%), SD 7 (29%), PD 2 (8%) and NE 4. Median survival is 7 months for regime A and 11 months for regime B ($P=0.19$). Survival is superior for 22 LD patients (LD *vs.* ED $P<0.05$). There have been 5 deaths in which toxicity may have been contributory (4 with regime A, 1 with regime B). Nausea and vomiting is grade 2 for regime A and grade 3 for regime B. The benefit of thoracic irradiation in CR is being assessed in a second randomisation. So far there are important differences in the toxicity profiles of the two regimes but no significant differences in response or survival. Stage is an important prognostic factor.

**Accurate dosimetry for targeted radionuclide therapy**

S.E. Dewhurst, S.J. Riggs, A.J. Green, R.H.T. Begent & K.D. Bagshawe

*Cancer Research Campaign Laboratories, Department of Medical Oncology, Charing Cross and Westminster Medical School, London W6 8RF, UK.*

The development of targeted therapy of cancer requires that the distribution of the therapeutic agent in tumour and normal tissues is reliably estimated. Gamma camera imaging has the potential to achieve this. However, present methods cannot accurately quantify radioactivity in sites with significant activity in tissues overlying those being studied. A method has been developed using single photon emission tomography (SPET) with correction for Compton scatter (Jaszczak *et al.*, 1984, *J. Nucl. Med.*, **25**, 893) to permit accurate measurement of activity in tumour and normal tissues of patients receiving $^{131}$Iodine labelled antibody. An IGE Gemini gamma camera with 400 keV collimator was used to acquire tomographic images of a phantom containing sources with levels of radioactivity similar to those found in human tumours in a background of lower levels of activity. The background was increased in increments of 0.2 from 0 to 1.2 $\mu Ci\,ml^{-1}$. Scatter corrected reconstructions gave accurate estimates of activity in the source regardless of background activity. Without correction there was an apparent rise in activity of 60% in the source. With planar reconstruction apparent activity in the source rose by up to 560%. The validity of the method was confirmed in patients by comparing activity in the cardiac ventricles measured by SPET with scatter correction with that in a simultaneous blood sample. A coefficient of correlation of 0.955 was achieved with 25 data points. SPET with scatter correction was compared with planar imaging in measuring activity in the liver and spleen of patients receiving 75 mCi $^{131}$I-antibody to CEA intravenously. Planar imaging gave significantly higher values than SPET for the spleen ($t=5.4$, $P<0.001$ by the paired $t$ test) but no significant difference for the liver. This shows the overestimation of activity by planar imaging structures like spleen or tumour which is overlaid by other tissues. SPET with scatter correction forms a basis for an improved technique of quantifying the targeting efficiency.

**The distribution and dosimetry of radiolabelled antibody in radioimmunotherapy**

R.H.J. Begent, J.A. Ledermann, A.J. Green, S.J. Riggs, F. Searle, T. Adam, P.A. Keep & K.D. Bagshawe

*Cancer Research Campaign, Labs, Charing Cross Hospital, London W6 8RF, UK.*

Antibody targeted therapy of cancer requires that a favourable distribution of antibody is sustained in tumour relative to normal tissues. The necessary data showing the time course of antibody distribution in man are lacking. The distribution of 40–150 mCi of intravenously administered 131-iodine labelled antibody to carcinoembryonic antigen has been studied in 16 patients with colorectal cancer. The distribution of antibody was measured by gamma camera imaging using single photon emission tomography with scatter correction (Riggs *et al.*, 1988, *Int. J. Cancer*, suppl. 2, **95**, 1988) and planar imaging as appropriate. Maximum concentrations of radioactivity were found in tumour 8 hours after administration. Activity in tumour varied up to 9-fold in different patients. Higher levels were found on average in tumour than any other tissue. Liver, lung and blood were the other tissues in which antibody was concentrated relative to the rest of body. Antibody cleared from all these tissues over 1 week. Second antibody directed against the antitumour (first) antibody was given 24 hours after first antibody in order to accelerate clearance from the blood. This increased the tumour to blood ratio but had little effect on other tissues. Cumulative radiation dose to tumour and normal tissue was estimated and showed that in patients with the most efficient localisation the tumour to body ratio was 20:1 and tumour to blood ratio 5:1. This may be sufficient for effective therapy of cancer in patients selected for efficient antibody localisation.

**Breast cancer immunoscintigraphy with $^{123}$I and $^{125}$I-labelled monoclonal antibody (Mab) SM3**

C. Twelves[1], S. Clarke[1], A. Girling[1], C. Lazarus[1], S. Allen[1], J. Taylor[2], J. Burchell[1], S. Mather[3], M.A. Richards[1] & R.D. Rubens[1]

[1]*ICRF Clin. Oncol. Unit, and Div. Radiobiological Sciences, Guy's Hospital, London SE1;* [2]*ICRF Labs, London WC2, and* [3]*St Bartholomew's Hospital, London EC1, UK.*

A mouse antibody, SM3, has been evaluated in breast cancer patients undergoing mastectomy with axillary clearance. SM3 was labelled with $^{123}$I to assess early (24 h) or $^{125}$I to assess late (72 h) uptake. Labelled SM3 was injected either subcutaneously (s.c.) into the finger web spaces of both hands, or intravenously (i.v.). With $^{123}$I-SM3, patients were injected 24 h before surgery, imaged preoperatively, and the resected specimen imaged postoperatively. Radioactivity was counted in the surgical specimen and it was examined histologically. With $^{125}$I-SM3, the patients underwent mastectomy 72 h after injection, the surgical specimen was examined histologically and radioactivity counted. Images cannot be obtained with $^{125}$I.

Fourteen patients have been studied. Imaging with $^{123}$I-SM3 showed symmetrical axillary uptake and uptake in resected lymph nodes. The images did not correspond to histologically proven metastases, and the primary tumour was not visualised. Radioactivity in the surgical specimens is shown in the table as mean % injected $^{123}$I or $^{125}$I g$^{-1}$ tissue.

| | $^{123}$*I-SM3* (*s.c.*) $n=4(24\,h)$ | $^{123}$*I-SM3* (*i.v.*) $n=4(24\,h)$ | $^{125}$*I-SM3* (*i.v.*) $n=2(72\,h)$ | $^{125}$*I-SM3* (*i.v.*) $n=4(72\,h)$ |
|---|---|---|---|---|
| Normal nodes | 0.007 | – | 0.0066 | – |
| Metastatic nodes | 0.005 | – | 0.036 | – |
| Normal tissue | – | 0.005 | – | 0.0007 |
| Primary tumour | – | 0.0037 | – | 0.0024 |

This is a valuable model for evaluating immunoscintigraphy in breast cancer. We have not shown tumour-specific uptake of SM3 in lymph nodes or primary tumour 24 h after injection. However, preliminary data with $^{125}$I-SM3 suggests specific uptake may be detectable at 72 h.

**$^{131}$I mIBG absorbed dose estimates in neuroblastoma: calculations based on tumour-biopsy data**

J.W. Babich, J.S.E. Moyes & S.L. Fielding

*Royal Marsden Hospital and Institute of Cancer Research, Sutton, Surrey, UK.*

In an effort to understand more fully the potential role of $^{131}$I mIBG in the treatment of neuroblastoma, a study was undertaken to determine the quantitative tumour uptake of this radiopharmaceutical. Six children with neuroblastoma, 1 with ganglioneuroma and 1 with ganglioneuroblastoma were injected i.v. with $^{125}$I mIBG, 24–48 h prior to surgical removal of the tumour. Percent injected dose per gram of tumour was calculated and histopathology performed on all surgical specimens. The mean range of uptake in these tumour specimens were 0.0022–0.2% injected dose g$^{-1}$. A wide range of intra- and inter-tumour variation of mIBG uptake was demonstrated which was related to tumour pathology.

Absorbed dose estimates have been calculated for this range of tumour uptake for various activity-residence times and tumour masses, similar in magnitude to patient data. Based on these data, tumour dose estimates were in the range 0.11–10.4 Gy GBq$^{-1}$ (effective half-life, $T$, of 24 h), to 0.45–41.8 Gy GBq$^{-1}$ ($T=96$ h). From these calculations, it seems likely that certain patients will receive sterilising radiation doses to tumours and therefore a suitable means of selecting patients for therapy is required. In addition, microdosimetry must be evaluated in order to determine the absorbed dose to small tumour masses.

**Localised $^{31}$P magnetic resonance (MR) spectroscopy in patients with cerebral gliomas**

C.J. Twelves[1], D. Porter[1], M. Lowry[1], M.A. Smith[1], P. Heasley[1], P. Garlick[1], A. Moore[2], A. Bell[2], M. Maisey[1], R.D. Rubens[1] & M.A. Richards[1]

[1]*ICRF Clinical Oncology Unit, and Div. Radiological Sciences, Guy's Hospital and* [2]*Atkinson Morley's Hospital, London, UK.*

We have obtained localised $^{31}$P brain spectra from 15 patients with primary brain tumours >3 cm diameter and from 17 healthy volunteers with a 1.5 Tesla Philips imaging and spectroscopy system. Spectra were acquired using image selective *in vivo* spectroscopy (ISIS) from regions identified on MR images. The volume of brain studied was 125 cc for each volunteer. In 8 untreated glioma patients the tumour was studied, and in 6 glioma patients brain tissue outside the tumour was studied. The following peaks were identified: phosphomonoesters (PME), inorganic phosphate (Pi), phosphodiesters (PDE), phosphocreatine (PCr), $\gamma$-, $\alpha$- and $\beta$-ATP. Tissue pH was calculated from Pi-PCr chemical shift, Peak areas were expressed as % of total $^{31}$P signal, PCr/Pi, PCr/$\beta$-ATP and PDE/PME peak area ratios were calculated.

$^{31}$P spectra from brain tissue outside the tumour in glioma patients did not differ significantly from spectra from volunteers. All spectra collected from gliomas were abnormal. Mean values which differed significantly (Wilcoxon's rank sum test) from those in healthy volunteers are shown below.

| | *pH* | *Pi* | *PDE* | *PCr/Pi* |
|---|---|---|---|---|
| Volunteers ($n=7$) | 7.00 | 6.75 | 30.8 | 2.46 |
| Glioma ($n=8$) | 7.13 | 9.51 | 26.2 | 1.78 |
| *P* value | <0.01 | <0.05 | <0.01 | <0.01 |

These results show differences in bioenergetics (Pi and PCr/Pi), pH and membrane metabolism (PDE) between gliomas and normal brain. Similar abnormalities were observed in a patient with relapsed medulloblastoma. These resolved within 2 weeks of starting radiotherapy, before tumour size changed.

***In vivo* $^{31}$P nuclear resonance spectroscopy of human breast carcinoma: the importance of localisation**

J. Glaholm, J.C. Sharp, D.J. Collins, V.R. McReady, A. Hind & M.O. Leach

*The Royal Marsden Hospital and Institute of Cancer Research, Sutton, Surrey, UK, and Siemens Ltd, Sunbury on Thames, UK.*

*In vivo* $^{31}$P nuclear magnetic resonance spectroscopy provides for non-invasive assessment of the response of tumour metabolism to therapeutic intervention. Early prediction of treatment response and disease relapse are of considerable clinical importance.

The anatomical proximity of the breast to underlying skeletal muscle necessitates an accurate method of volume localisation in order to prevent spectral contamination by the strong signal from muscle. We have demonstrated the efficacy of a modified ISIS localisation sequence and the inadequacy of surface coil 'localisation'.

Six patients with locally advanced breast carcinoma have been assessed during treatment. Four were treated with combination chemotherapy, 1 with tamoxifen and 1 with external beam irradiation. Two patients responding to chemotherapy demonstrated spectral changes, in one case these preceded the clinical response. The tamoxifen treated patient similarly demonstrated spectral changes, however these were not shown to precede clinically measurable changes. The patient treated with external beam irradiation showed general signal reduction which parallelled tumour volume reduction, however surprisingly there was no qualitative spectral change.

**Cellular recovery in two lines of the L5178Y murine lymphoma which differ in their sensitivity to ionising radiation**

T.J. McMillan, J.J. Eady, J.H. Peacock & G.G. Steel

*Radiotherapy Research Unit, Institute of Cancer Research, Sutton, Surrey, UK.*

Two lines of the murine lymphoma, L5178Y, have been investigated to assess the role of cellular recovery in the determination of their widely different sensitivities to ionising radiation. Two commonly used assays of recovery capacity have been used. The extent of dose-rate sparing is measured by comparing the survival of the cells after acute and low dose rate irradiation and split dose experiments measure the extent of recovery when two halves of an acute dose are separated in time.

LY-R seems to have the greater dose-rate effect when the dose reduction factor at a survival of 0.01 is used (1.65 and 1.37 for LY-R and LY-S respectively), suggesting that its recovery capacity is greater.

Split dose experiments do not, however, confirm this. Classically split dose measurements have been made at a single dose level which is chosen to give a selected level of survival (often 0.01) but we have shown that this is inadequate because recovery ratio (RR) increases with dose. The linear quadratic equation determines that this increase in RR with dose is a function of the constant $\beta$ so that $\beta$ gives a better indication of recovery capacity. Making this comparison between LY-R and LY-S we come to the conclusion that the most sensitive line has in fact the greater recovery capacity.

It is apparent, then, that the assessment of recovery capacity is not straightforward and careful analysis is required before connections are made between recovery and cellular sensitivity.

**Split-dose recovery reassessed**

J.H. Peacock, T.J. McMillan & G.G. Steel

*Radiotherapy Research Unit, Institute of Cancer Research, Sutton, Surrey, UK.*

In a recent study of the effect of dose on split-dose recovery in four human tumour cell lines we suggested that an increased capacity for cellular recovery did not necessarily correlate with increased cellular radiosensitivity. We have now extended this to a total of 16 normal and tumour cell lines, including several highly radiosensitive human A-T lines. In addition we have reanalysed previously published data where the effect of dose has been studied.

Analysis of the results for these lines indicate that: (i) All lines demonstrate increasing split-dose recovery with increasing dose. This agrees well with the relationship predicted by the linear-quadratic equation (equation 1), and enables us to use the $\beta$ value as a measure of recovery:

$$\ln(\text{Recovery Ratio}) = 2\beta d^2 \qquad (1)$$

where $d$ is dose per fraction and $\beta$ is from the L-Q equation. (ii) At any given dose split-dose recovery was greatest in the most radiosensitive cells. Analysis using equation (1) yields values for $\beta$ that increase with increasing radiosensitivity.

The results presented here reinforce our earlier conclusion that the concept of radiosensitive cells being recovery deficient is wrong. The importance of this conclusion lies in the possible relationship between recovery and repair. It is generally assumed that capacity for cellular recovery reflects the ability to repair radiation-induced sub-cellular damage. If this assumption is true then our results suggest that increasing cellular radiosensitivity does not result from a decreased ability for repair.

**The effects of three bioreductive drugs, mitomycin C, RSU-1069 and SR-4233 on cell lines selected for their sensitivity to DNA-damaging agents**

A. Keohane, J. Godden, I.J. Stratford & G.E. Adams

*MRC Radiobiology Unit, Chilton, Didcot, Oxon OX11 0RD, UK.*

We have investigated the cross-sensitivity of a number of cell lines to mitomycin C, RSU-1069 and SR-4233 under both aerobic and hypoxic conditions. The cell lines used were selected for their sensitivity to DNA-damaging agents and fall into two groups. The first, MMC cells (Robson *et al.*, 1985, *Cancer Res.*, **45**, 5304) were derived from CHO-K1 cells and show a range of sensitivities to mitomycin C in air ($D_{37}$ values ($\mu g\,ml^{-1}$) after 3 h aerobic incubation are: K1, 0.65; MMC-2, 0.25; MMC-3, 0.17; MMC-R, 8.0). The second group of cells cloned from V79 fibroblasts (Jones *et al.*, 1987, *Mutation Res.*, **183**, 279) exhibit sensitivity to ionising radiation ($D_{37}$ values (Gy) of these cells are: V79, 4.2; $irs_1$, 1.34; $irs_2$, 1.41; $irs_3$, 2.06). No difference in the aerobic or hypoxic toxicity of mitomycin C was observed for the CHO-K1, MMC-2 or MMC-3 cells. However, the MMC-resistant cell line (MMC-R) was 10 times more sensitive under hypoxic ($D_{37}$ 0.8 $\mu g\,ml^{-1}$) than aerobic conditions. This suggests that MMC-R cells lack or have a lower expression of the $O_2$-*insensitive* reductase system compared to the other MMC cells. In contrast, all CHO cell lines showed greater sensitivity to RSU-1069 and SR-4233 under nitrogen compared to air, with differential toxicities between 3 and 30. There was little or no cross-sensitivity when compared to the responses to MMC in either air or $N_2$. Treatment of V79 and irs cells with RSU-1069 and SR-4233 also resulted in selective toxicity towards hypoxic cells. Differential toxicities between 50 and 100 were observed in wild type V79s. For both drugs the hypoxic toxicities were similar in both the parent and irs cells, but in air the mutant cells were up to 10 times more sensitive. (Supported by NCI grant no. R01CA44 126-01.)

**Bioreductive drugs and the selective induction of tumour hypoxia**

J.C.M. Bremner, I.J. Stratford & G.E. Adams

*MRC Radiobiology Unit, Chilton, Oxon OX11 0RD, UK.*

The hypoxic fraction present in most experimental tumours provides the possibility of selective cell killing by bioreductive agents. Increasing tumour hypoxia should further enhance the efficacy of these agents. Methods of selectively increasing tumour hypoxia to close to 100% include clamping or by use of the drugs: hydralazine (5 mg $kg^{-1}$), or flavone acetic acid, FAA, (200 mg $kg^{-1}$). We have measured their effectiveness in potentiating the bioreductive toxicity of: RSU 1069 (80 mg $kg^{-1}$), misonidazole (800 mg $kg^{-1}$), mitomycin C (5 mg $kg^{-1}$) and SR 4233 (50 mg $kg^{-1}$) in KHT and RIF-1 tumours grown subcutaneously in C3H mice. Response to treatment was measured as the time for tumours to reach 4 × initial treatment volume. For untreated controls this was 3.58 ± 0.26 and 5.19 ± 0.29 days respectively.

Clamping alone for 90 min gave little or no response, but when RSU 1069 is administered 15 min prior to clamping, large growth delays result in KHT and RIF-1 tumours (17.35 ± 0.49 and 27.52 ± 2.08 days respectively). RIF-1 tumours clamped for 120 min with RSU 1069 gave 30% tumour control with no recurrence at 150 days. Less effect was seen with misonidazole, mitomycin C and SR 4233 in the KHT tumour (3.63 ± 0.33, 7.90 ± 1.23 and 7.90 ± 0.49 days respectively). Hydralazine showed some growth delay with RSU 1069 in the RIF-1 (9.28 ± 0.36) but no effect in the KHT tumour. FAA alone gives growth delay in the KHT and RIF-1 tumours (9.53 ± 1.86 and 8.37 ± 1.59 days), but the addition of 1069 provides little or no increase in this effect. These results indicate that induction of tumour hypoxia together with a bioreductive drug can be an effective approach to therapy. However, the results also imply that the depth and duration of tumour hypoxia will contribute to the effectiveness of the bioreductive drug under examination.

The organisers gratefully acknowledge the generous support of Merck, Sharp & Dohme, who partially sponsored the meeting, and the Cancer Research Campaign and Imperial Cancer Research Fund, who each sponsored one of the overseas speakers. In addition, the meeting has been kindly supported by the following companies: Bristol-Myers, Book Show, Lederle Laboratories and Wise Press.